# Integrated Metabolomics and Machine Learning Reveal Drying‐Dependent Phenolic Markers in Asteraceae Edible Plants From Ulleungdo Island

**DOI:** 10.1002/fsn3.72297

**Published:** 2026-09-02

**Authors:** In Young Lee, Doo‐Hee Lee, Ju Hong Park, Nami Joo

**Affiliations:** ^1^ Department of Convergence IT Engineering Pohang University of Science and Technology (POSTECH) Pohang Republic of Korea; ^2^ National Instrumentation Center for Environmental Management Seoul National University Seoul Republic of Korea; ^3^ Department of Food and Nutrition Sookmyung Women's University Seoul Republic of Korea

**Keywords:** Asteraceae, explainable machine learning, Ulleungdo wild vegetables, untargeted metabolomics

## Abstract

Ulleungdo Island, Korea, features a distinctive oceanic climate that supports a diverse array of edible wild plants traditionally processed through blanching and sun‐drying. Despite their cultural and commercial significance, the effects of traditional drying methods on phytochemical composition remain inadequately understood. This study investigated processing‐induced metabolic changes in three representative Asteraceae species—*Aster pseudoglehnii*, *Cirsium nipponicum*, and *Solidago virgaurea*—using integrated metabolomics and machine learning approaches. Untargeted UHPLC–HRMS/MS profiling identified 110 secondary metabolites across freeze‐dried and sun‐dried samples. Hierarchical clustering revealed that species identity was the primary determinant of chemotype, while processing induced consistent quantitative shifts within each species. Traditional processing (blanching followed by sun‐drying) significantly reduced photo‐ and enzymatically labile flavonoid glycosides, whereas caffeoylquinic acid derivatives were relatively preserved. Random Forest analysis combined with SHAP interpretation identified caffeoylquinic acids, including chlorogenic acid and dicaffeoylquinic acid isomers, as processing‐stable chemotaxonomic markers, while glycosylated flavonoids served as sensitive indicators of processing intensity. Targeted quantification confirmed these trends and revealed species‐specific phenolic allocation patterns. Overall, this study demonstrates that integrating metabolomics with explainable machine learning enables robust identification of processing‐dependent marker compounds and provides a biochemical foundation for traditional drying practices and marker‐based quality control strategies for Ulleungdo wild vegetables.

## Graphical abstract

Three edible Asteraceae plants (*Cirsium nipponicum*, *Solidago virgaurea*, and *Aster pseudoglehnii*) from Ulleungdo Island, Korea, were processed by freeze‐drying or traditional blanching and sun‐drying. Untargeted UHPLC–HRMS metabolomics (110 features) combined with targeted quantification of 33 phenolic compounds revealed distinct drying‐induced metabolomic shifts. Random Forest classification and SHAP analysis identified 19 shared biomarker candidates—including rutin, 4‐O‐coumaroylquinic acid, and eriodictyol 7‐O‐malonylglucoside—that discriminate drying treatments, with chlorogenic acid as the most abundant phenolic across all samples.

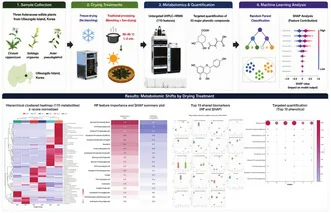

## 1 Introduction

Plant secondary metabolites, including phenolic acids, flavonoids, tannins, and terpenoids, play crucial roles in plant adaptation and influence the nutritional and functional properties of edible species (Dewick 2002; Grace and Logan 2000). These bioactive compounds are synthesized via the shikimate–phenylpropanoid–flavonoid pathways and are highly responsive to environmental factors such as temperature, light intensity, and oxidative stress (Davies et al. 2020; Nakabayashi and Saito 2015). Plants growing in geographically isolated or environmentally stressed ecosystems often accumulate distinctive metabolite profiles that serve important ecological and physiological functions (Brunetti et al. 2013; Fernández‐Marín et al. 2020; Lee et al. 2025).

Ulleungdo Island, a volcanic island located in the East Sea of Korea, features a unique oceanic climate characterized by high humidity, mineral‐rich volcanic soils, and notable isolation from the mainland. These environmental conditions have fostered a diverse flora comprising over 500 vascular plant species, including numerous endemic taxa (Chung et al. 2020; Sampaio et al. 2016), making Ulleungdo Island a natural laboratory for investigating how isolation and environmental stress influence secondary metabolite diversity (Brunetti et al. 2013).

Despite the widespread consumption and commercial distribution of edible wild vegetables (sanchae) from Ulleungdo Island, scientific understanding of how traditional post‐harvest processing affects their bioactive compound composition remains limited. Local residents have long practiced a specific method in which freshly harvested plants are briefly blanched in boiling water and then sun‐dried to enhance shelf life and flavor while preserving the product's appearance (Chan et al. 2009). However, these thermal and photochemical processes may significantly alter phenolic and terpenoid metabolites through oxidation, polymerization, or enzymatic inactivation (Dewanto et al. 2002; Moyo et al. 2018; Mbondo et al. 2018; Sultana et al. 2012; Mediani et al. 2015). Although there is growing interest in terroir‐based functional foods, no comparative metabolomic analysis has been conducted on fresh versus traditionally processed sanchae from Ulleungdo Island.

The present study focuses on three representative edible species native to Ulleungdo Island: *Aster pseudoglehnii* (Seombujikkaengi), *Solidago virgaurea* (Miyeokchwi), and *Cirsium nipponicum* (Muleonggeongkwi). These species belong to the family Asteraceae (order Asterales). Plants in the Asteraceae family are known for their abundant sesquiterpene lactones and caffeoylquinic acids (CQA) (Awuchi and Morya 2023; Rolnik and Olas 2021; Nalewajko‐Sieliwoniuk et al. 2019; Gouveia and Castilho 2011; Clifford et al. 2006). Comparative profiling of these taxa is therefore expected to reveal both conserved and lineage‐specific responses to heat and sunlight exposure, thereby contributing to the understanding of post‐harvest metabolic transformations and the chemical basis underlying traditional processing practices (Akhatou et al. 2017).

To elucidate these differences, an untargeted metabolomics approach based on UHPLC–HRMS/MS was employed to profile and quantify phytochemicals in both freeze‐dried and traditionally sun‐dried samples (Dunn et al. 2011; Gilbert‐López et al. 2017). This analytical platform enables high‐sensitivity detection of a broad spectrum of secondary metabolites, including phenolic acids and flavonoids, which are often linked to antioxidant potential and plant defense mechanisms (Kind et al. 2009; Johnson et al. 2016). Previous studies have primarily focused on monitoring changes in total phenolic content or simple antioxidant activities using conventional statistical methods (Sultana et al. 2012; Shofian et al. 2011; Turkmen et al. 2005). However, these approaches are limited because metabolomic data are inherently high‐dimensional and nonlinear, making it difficult for traditional multivariate methods to identify reliable discriminant markers (Gromski et al. 2014). Recent advances in food metabolomics have demonstrated the effectiveness of machine learning techniques—particularly Random Forest (RF) combined with SHAP (SHapley Additive exPlanations)—in resolving complex metabolic interactions and enhancing the interpretability of feature importance (Huang et al. 2023; Chen et al. 2013; Luo et al. 2024). These explainable AI methods provide robust insights into compositional shifts associated with processing treatments and have emerged as valuable tools for biomarker discovery in plant‐based foods (Xiao et al. 2025).

Therefore, this study aims to comprehensively evaluate the metabolomic variations in three representative Asteraceae edible plants native to Ulleungdo Island under two distinct processing conditions: traditional drying and freeze‐drying. We applied an integrated metabolomics strategy that combines untargeted UHPLC‐HRMS profiling for broad screening with targeted quantitative analysis for precise verification. Specifically, we utilized a Random Forest‐based feature selection method coupled with SHAP analysis to identify key phenolic markers that differentiate processing methods and plant species. Finally, the selected markers were quantified to provide a scientific basis (Ibáñez et al. 2015) for optimizing the processing of Ulleungdo's biological resources. This comparative metabolomics approach is expected to elucidate qualitative and quantitative changes in secondary metabolites induced by blanching and sun‐drying processes, confirm the stability of phenolic compounds in Asteraceae plants, and clarify the biochemical basis of traditional processing methods in the Ulleungdo terroir. Through this integrated approach, the present study provides a mechanistic and analytical foundation for understanding traditional processing effects and enhancing the functional value of regionally distinctive wild vegetables.

## 2 Materials and Methods

### 2.1 Reagents and Standards

LC–MS‐grade acetonitrile, methanol, and water containing 0.1% formic acid were purchased from Fisher Scientific (Pittsburgh, PA, USA). Analytical standards of 8 hydroxycinnamic acids (3‐*O*‐coumaroylquinic acid, 3‐*O*‐feruloylquinic acid, 3,5‐Di‐caffeoylquinic acid, 4‐*O‐*coumaroylquinic acid, 5‐*O*‐feruloylquinic acid, 5‐D‐caffeoylquinic acid, Cryptochlorogenic acid, Ferulic acid), 2 flavanones (Eriodictyol, Naringenin), 8 flavones (Apigenin, Apigenin 6,8‐C‐diglucoside (Vicenin 2), Apigenin 6‐C‐glucoside, Chrysoeriol, Diosmetin, Luteolin, Luteolin 7*‐O‐*glucoside, Scutellarein), 13 flavonols (Isorhamnetin, Isorhamnetin 3*‐O‐*rutinoside, Kaempferide, Kaempferol, Kaempferol 3*‐O‐*glucoside, Kaempferol 3*‐O‐*rhamnoside, Kaempferol‐3*‐O‐*rutinoside, Kaempferol‐3‐sambubioside, Quercetin, Quercetin‐3*‐O‐*glucoside, Quercetin‐3*‐O‐*malonylglucoside, Quercetin‐3*‐O‐*rhamnoside, Quercetin‐3*‐O‐*rutinoside) and 2 Flavanols (Catechin, Epicatechin) were purchased from Sigma‐Aldrich (St. Louis, MO, USA), Biofron (La Mirada, CA, USA) and LGC Standards (Wesel, Germany).

### 2.2 Plant Materials and Sampling

Three edible plant species native to Ulleungdo Island, Korea—*Aster pseudoglehnii* (*A. pseudoglehnii*), *Solidago virgaurea* (*S. virgaurea*), and *Cirsium nipponicum* ( *C. nipponicum* )—were selected for comparative metabolomic analysis. Fresh aerial parts (young leaves and stems) were collected from natural habitats on Ulleungdo Island (37°50′–37°52′ N, 130°85′–130°88′ E) on three independent sampling occasions between April 5 and April 20, 2024, providing three biological replicates (*n* = 3) for each species. Immediately after harvest, each biological sample was divided into two portions to ensure that both drying treatments originated from the same field‐collected sample. One portion was freeze‐dried for at least 72 h using a freeze dryer (Ilshin Biobase, Dongducheon, Korea), producing freeze‐dried samples of *A. pseudoglehnii* (FAP), *C. nipponicum* (FCN), and *S. virgaurea* (FSV). The second portion was processed using a traditional sun‐drying method commonly practiced on Ulleungdo Island. Specifically, fresh materials were blanched in boiling water (90°C–95°C) for 1–2 min and then sun‐dried for 2–3 days at ambient temperatures (18°C–25°C) under natural sunlight with continuous air circulation, resulting in sun‐dried samples of *A. pseudoglehnii* (SAP), *C. nipponicum* (SCN), and *S. virgaurea* (SSV). All dried samples were ground into a fine powder using a cryogenic grinder (Retsch MM400, Germany) and then passed through a 60‐mesh sieve prior to metabolomic analysis. Each biological sample was extracted independently in triplicate, and the resulting data were used for subsequent metabolomic, statistical, and machine‐learning analyses.

### 2.3 Extraction of Metabolites

Each powdered sample (100 mg) was extracted with 1.0 mL of 80% methanol (v/v, LC–MS grade) containing 0.1% formic acid. The mixture was vortexed for 1 min, sonicated for 30 min at room temperature, and centrifuged at 12,000×*g* for 10 min at 4°C. The supernatant was filtered through a 0.22 μm PTFE syringe filter and transferred to amber vials. Extraction was performed in triplicate for each independently field‐collected biological replicate. The combined filtrates were used for both UHPLC–HRMS profiling and targeted quantification analyses.

### 2.4 UHPLC–HRMS Profiling

Plant extracts were subjected to non‐targeted metabolite profiling using ultra‐high‐performance liquid chromatography coupled with high‐resolution Orbitrap mass spectrometry (UHPLC–Orbitrap–HRMS). Chromatographic analysis was conducted on a Vanquish Flex UHPLC system (Thermo Fisher Scientific, MA, USA) equipped with a reversed‐phase CORTECS T3 column (2.1 × 150 mm, 1.6 μm particle size, 120 Å pore size; Waters, Milford, USA). The column oven was maintained at 45°C, and analytes were eluted at a constant flow rate of 0.25 mL/min with an injection volume of 1 μL. The chromatographic system utilized a binary solvent system comprising mobile phase A (0.1% formic acid in LC–MS grade water) and mobile phase B (0.1% formic acid in acetonitrile). Separation was achieved using a multistep gradient program, beginning with 3% B held for 0.5 min, followed by a gradual increase to 15% B over 15.0 min, a further rise to 95% B by 50.0 min, with an isocratic hold at 95% B until 55.0 min. The gradient was subsequently returned to the initial conditions within 1.0 min and equilibrated at 5% B until 60.0 min.

Mass spectrometric detection was performed using a Q Exactive Plus Orbitrap mass spectrometer (Thermo Fisher Scientific, MA, USA) operated in both positive and negative electrospray ionization modes. Full‐scan acquisition was combined with data‐dependent MS/MS (ddMS^2^) analysis over an m/z range of 100–1500. The resolving power was set to 70,000 for full MS scans and 17,500 for MS/MS scans. The heated ESI source was configured with sheath, auxiliary, and sweep gas flow rates of 45, 10, and 1 arbitrary units, respectively; capillary voltages of 3.8 kV (positive) and 3.2 kV (negative); and ion transfer tube and capillary temperatures maintained at 350°C.

Raw data were acquired and processed using TraceFinder software (version 4.1; Thermo Fisher Scientific). Non‐targeted metabolite annotation and profiling were performed using Compound Discoverer software (version 3.3; Thermo Fisher Scientific, Waltham, MA, USA). A comprehensive workflow for the Compound Discoverer analysis is presented in Table S1.

### 2.5 Machine Learning Analysis

To identify discriminant metabolites associated with species differences and drying methods, machine learning analysis was conducted using the metabolite intensity matrix obtained from untargeted UHPLC–HRMS profiling. A RF classifier was initially applied to evaluate the relative contribution of each metabolite to sample differentiation. The model was trained using 500 decision trees with the Gini impurity criterion and a “sqrt” feature selection strategy to prevent overfitting while maximizing classification performance. Model performance was assessed using leave‐one‐out cross‐validation (LOOCV) due to the limited sample size. Classification accuracy, macro‐averaged F1‐score, and one‐versus‐rest ROC–AUC values were calculated, and confusion matrices were generated to visualize classification outcomes. Feature importance values were extracted based on the mean decrease in impurity, enabling the identification of metabolites that most strongly contributed to distinguishing freeze‐dried from sun‐dried samples across the three Asteraceae species. Given the limited sample size (*n* = 18) relative to the number of features (110), a permutation (label‐shuffling) test was additionally performed to evaluate whether the observed classification accuracy exceeded chance expectation: class labels were randomly permuted 1000 times, the identical RF–LOOCV pipeline was re‐fitted on each permutation, and an empirical *p*‐value was calculated by comparing the observed accuracy to the resulting null distribution. In parallel, model stability was assessed using repeated stratified *k*‐fold cross‐validation (3‐fold, repeated 100 times) and repeated held‐out validation (100 random splits, with one sample per class held out in each split).

To enhance interpretability and quantify the local influence of individual metabolites on model predictions, SHAP (SHapley Additive exPlanations) values were computed using the TreeExplainer framework. SHAP summary plots visualized the magnitude and direction of each metabolite's impact on the classification outcome. By comparing RF feature importance with SHAP values, a Δ (RF—SHAP) metric was calculated to distinguish robust, processing‐stable metabolites from those sensitive to drying‐induced alterations. Metabolites appearing in both the top 20 by RF Gini importance and the top 20 by mean absolute SHAP value were selected as candidate markers for subsequent targeted quantification. This integrated RF–SHAP workflow enabled transparent and biologically meaningful interpretation of machine‐learning results, overcoming the limitations of conventional multivariate analyses applied to high‐dimensional metabolomic datasets.

### 2.6 Targeted Quantification of Phenolic Metabolites

Targeted analysis of phenolic secondary metabolites was conducted following the protocol reported by Lee and Joo (2022) and Marksa et al. (2020), with appropriate modifications. Sample extracts prepared in 80% aqueous methanol were quantitatively analyzed using an Vanquish Flex UHPLC (Thermo Fisher Scientific) equipped with a reversed‐phase CORTECS C18 column (2.1 × 150 mm, 1.6 μm particle size, 120 Å pore size). The column temperature was maintained at 45°C, and chromatographic separation was carried out at a constant flow rate of 0.25 mL/min with an injection volume of 1 μL. The mobile phase consisted of solvent A (0.1% formic acid in LC–MS‐grade water) and solvent B (0.1% formic acid in LC–MS‐grade acetonitrile). Elution was performed using a programmed gradient, starting at 5% solvent B, which was increased to 25% over the first 1.0 min, followed by a linear increase to 100% solvent B over 10.0 min. The composition was held at 100% B for 0.5 min, then returned to the initial conditions and re‐equilibrated prior to the next injection.

Detection was performed using a triple quadrupole mass spectrometer (Thermo TSQ Altis) equipped with a Heated‐ESI II source, operated in polarity‐switching selected reaction monitoring (SRM) mode. Compound‐specific parameters—including retention times, ionization polarities, precursor‐to‐product ion transitions, and adduct forms—were optimized and are summarized in Table S2. The Heated‐ESI source conditions were set with capillary voltages of 3.5 kV in positive mode and 2.5 kV in negative mode, an ion transfer tube and capillary temperature of 320°C, and sheath, auxiliary, and sweep gas flow rates of 50, 10, and 1 arbitrary units, respectively. Data acquisition and quantitative processing were performed using TraceFinder software (version 5.0). Calibration curves were generated using five concentrations (1–100 ng/mL) of standard compounds (purity ≥ 98%, Sigma‐Aldrich) dissolved in methanol under identical chromatographic conditions (Figure S1). Linear regression analysis of the analytical peak area versus concentration yielded correlation coefficients (*R* ^2^) greater than 0.996 (Table S3). The limits of detection (LOD) and quantitation (LOQ) were determined based on signal‐to‐noise ratios of 3:1 and 10:1, respectively. Quality control (QC) samples were analyzed every 10 injections to monitor instrument performance.

### 2.7 Statistical Analysis

All quantitative data are presented as the mean ± standard deviation (SD) of three replicate measurements. Hierarchical clustering analysis (HCA, Ward linkage, and Euclidean distance) was performed with MetaboAnalyst 6.0 to visualize overall metabolic variation and identify discriminant markers. Quantitative results were analyzed by one‐way analysis of variance (ANOVA) to determine significant differences (*p* < 0.05) between plant species or processing conditions.

## 3 Results

### 3.1 Metabolite Identification

Untargeted UHPLC–HRMS/MS profiling identified 110 metabolites across six sample groups representing three Asteraceae species, processed either by freeze‐drying or traditional sun‐drying. The major chemical classes detected—including CQAs, coumaroylquinic acids, flavonol and flavone glycosides (Table S4, Figures S2 and S3)—are consistent with previous reports describing the phenolic richness and chemotaxonomic signatures of edible Asteraceae plants (Awuchi and Morya 2023; Rolnik and Olas 2021; Nalewajko‐Sieliwoniuk et al. 2019). These compounds were present in all samples, although their relative intensities varied markedly depending on the species and drying method.

**FIGURE 1:**
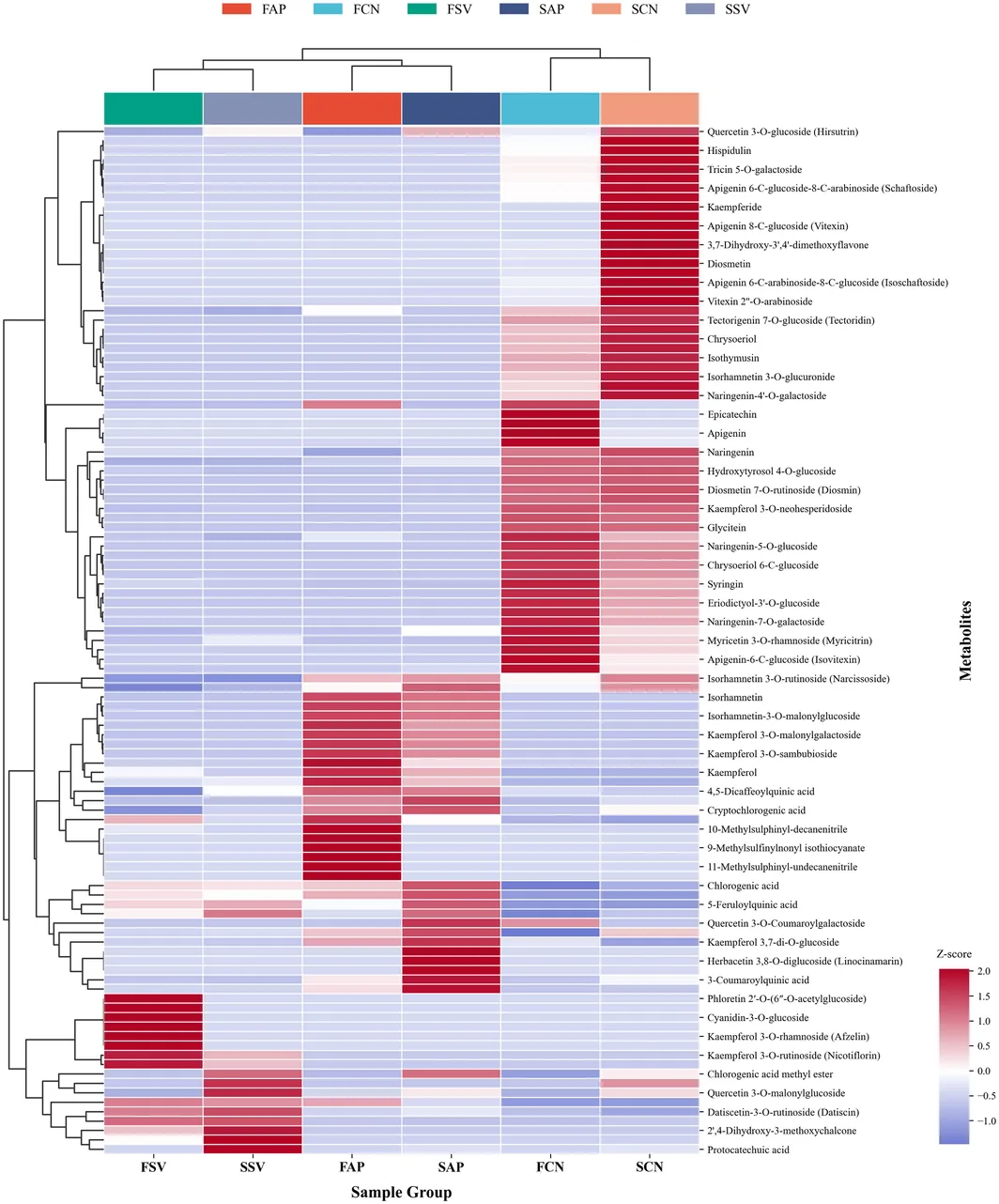
Hierarchical clustered heatmap showing the relative abundance (z‐score normalized) of metabolites detected in Asteraceae edible plants from Ulleungdo Island.

Hierarchical clustered heatmap analysis provided a detailed visualization of these patterns and revealed that species identity exerted the strongest influence on overall sample clustering (Figure 1). For each of the three Asteraceae taxa, freeze‐dried and sun‐dried samples grouped together before joining clusters of other species, indicating that the intrinsic chemotype of each plant was preserved regardless of processing conditions. This species‐dominant clustering aligns with earlier observations that Asteraceae plants maintain stable qualitative phenolic profiles primarily determined by genetic and biosynthetic factors (Gouveia and Castilho 2011; Marksa et al. 2020). However, within these species‐level branches, freeze‐dried and sun‐dried samples consistently separated into distinct subclusters, demonstrating that the drying method modulated quantitative patterns of phenolic subclasses while preserving species‐specific frameworks.

The heatmap revealed a highly consistent metabolic shift associated with traditional sun‐drying across all three species. Glycosylated flavonoids—including rutin, datiscetin‐3*‐O‐*rutinoside, and kaempferol derivatives—were significantly reduced in sun‐dried samples compared to their freeze‐dried counterparts. This decline is consistent with the susceptibility of flavonoid glycosides to prolonged photo‐oxidation and enzymatic deglycosylation during the 2–3‐day ambient‐temperature (18°C–25°C) sun‐drying step, with an additional contribution from the brief high‐temperature (90°C–95°C, 1–2 min) blanching pretreatment (Sultana et al. 2012; Turkmen et al. 2005). Conversely, CQA derivatives, particularly 3,5‐ and 4,5‐di‐ CQAs, were relatively enriched or maintained in sun‐dried samples. Their stability under drying conditions supports previous findings that these compounds exhibit high thermal robustness and may appear concentrated due to moisture loss (Dewanto et al. 2002; Moyo et al. 2018; Mbondo et al. 2018; Ratti 2001). The different responses of phenolic subclasses are likely attributable to their structural characteristics. Flavonoid O‐glycosides contain glycosidic bonds that are susceptible to hydrolysis and subsequent oxidation during prolonged exposure to heat, light, and endogenous enzymes. Once deglycosylated, the released aglycones become more vulnerable to oxidative degradation due to their highly reactive phenolic hydroxyl groups. In contrast, CQAs possess esterified hydroxycinnamic acid moieties that generally exhibit greater stability under moderate drying conditions, although prolonged heating may still induce isomerization or limited hydrolysis (Wianowska and Gil 2019). These structural differences provide a plausible explanation for the selective preservation of CQAs and the greater reduction of flavonoid glycosides observed after traditional processing.

Despite shifts induced by processing, each species retained distinct phenolic profiles. *C. nipponicum* consistently exhibited elevated levels of di‐CQAs; *S. virgaurea* was enriched in C‐glycosylated apigenin derivatives, such as schaftoside; and *A. pseudoglehnii* was characterized by higher levels of apigenin‐ and chrysoeriol‐type flavones. These species‐specific features, observed in both freeze‐dried and sun‐dried samples, underscore the predominant role of taxonomic factors in shaping metabolomic profiles, corroborating earlier phytochemical characterizations of Asteraceae taxa (Awuchi and Morya 2023; Rolnik and Olas 2021).

Taken together, the hierarchical clustering results demonstrate that species‐specific chemotypes primarily govern metabolomic organization, while the drying method induces consistent and predictable shifts within each species—reducing labile flavonoid glycosides and retaining or increasing thermally stable phenolic acids. This interpretation aligns with findings from earlier drying studies across various plant matrices and provides a clear biochemical rationale for subsequent machine learning–based marker identification.

### 3.2 Machine Learning–Based Determination of Potential Marker Compounds

To deepen the interpretation of the metabolomic patterns observed through multivariate analyses, machine learning models were employed to identify metabolites that most strongly contributed to sample differentiation. The application of RF to the full UHPLC–HRMS dataset revealed a clear ranking of metabolites based on their contribution to classification outcomes. The resulting feature‐importance distribution indicated that a subset of phenolic acids and flavonoid glycosides played disproportionately large roles in separating the six sample groups. In particular, chlorogenic acid and its di‐CQA derivatives consistently achieved the highest RF importance scores, reflecting both their species specificity and relative enrichment in sun‐dried samples. Similar findings have been reported in previous metabolomic studies of Asteraceae taxa, where di‐CQAs served as robust discriminants due to their structural stability and taxonomic specificity (Gouveia and Castilho 2011; Ianni et al. 2022). The prominent role of these metabolites in differentiating *Cirsium* species is therefore consistent with earlier phytochemical characterizations and reinforces the strong chemotaxonomic signal detected here (Awuchi and Morya 2023; Rolnik and Olas 2021).

Among the top‐ranked features, CQA derivatives—particularly chlorogenic acid and its di*‐O‐*CQA isomers (3,5‐ and 4,5‐di*‐O‐*caffeoylquinic acids)—consistently exhibited the highest RF importance scores. These compounds are well‐recognized chemotaxonomic markers in Asteraceae plants and are known for their structural stability under moderate thermal stress, which may contribute to their observed importance in distinguishing species and processing conditions within the present dataset. The prominence of these metabolites in the RF model aligns with previous studies demonstrating that CQA derivatives serve as robust markers in metabolomic classification tasks involving processing or geographical origin (Gouveia and Castilho 2011; Ianni et al. 2022).

**FIGURE 2:**
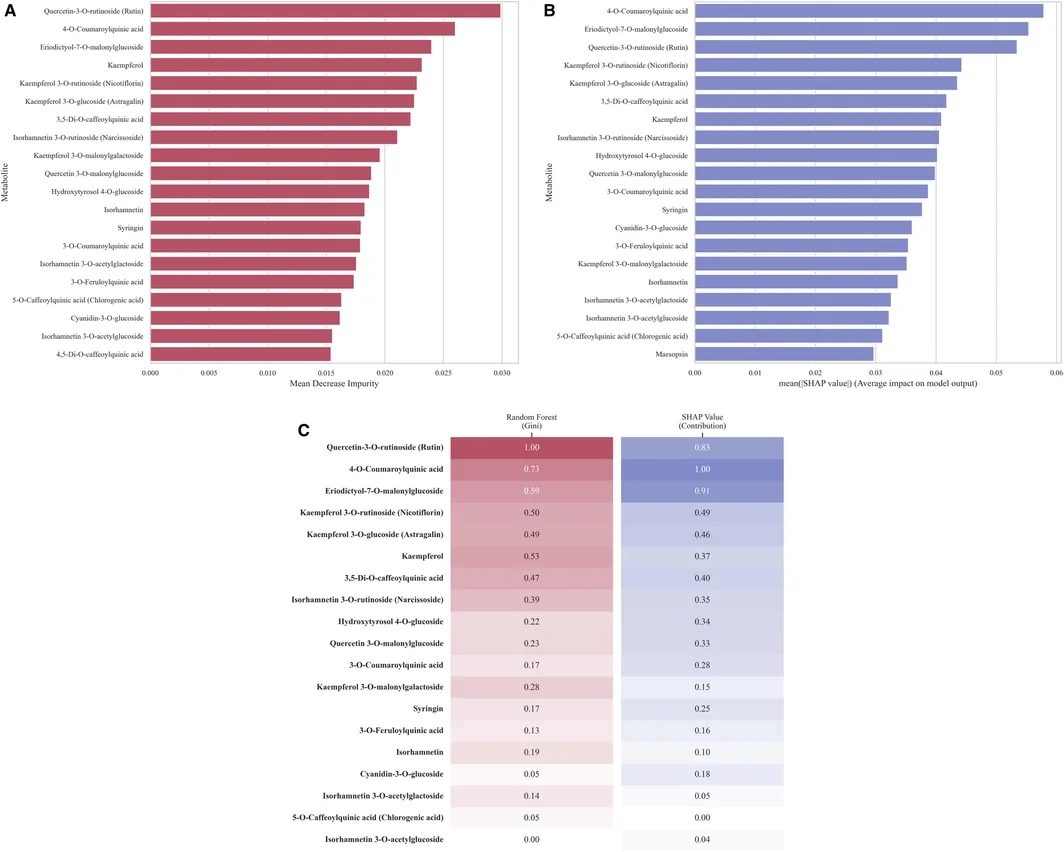
Random Forest–based feature importance and SHAP summary plot of metabolite profiles in *Asteraceae* edible plants from Ulleungdo Island. (A) Relative importance of metabolites derived from the Random Forest model based on the Gini index. (B) Contribution of individual metabolites to the classification model as determined by SHAP analysis. (C) Comparison of Random Forest feature importance and SHAP contribution values for the major metabolites.

To enhance model interpretability and evaluate the directionality of metabolite contributions, SHAP (SHapley Additive exPlanations) analysis was conducted on the trained RF model. The SHAP summary plot in Figure 2 illustrates how individual metabolites influence sample classification into freeze‐dried or sun‐dried groups. Glycosylated flavonoids, including rutin, kaempferol 3‐*O*‐rutinoside, and quercetin 3*‐O‐*malonylglucoside, exhibited strongly positive SHAP values for freeze‐dried samples and negative contributions for sun‐dried samples. This pattern reflects their higher abundance and preservation under low‐temperature dehydration, as well as their susceptibility to prolonged photo‐oxidative degradation and enzymatic deglycosylation during ambient‐temperature sun‐drying, together with a contribution from the brief high‐temperature blanching pretreatment. These findings are consistent with established degradation pathways of flavonoid glycosides reported under heat‐ and light‐exposed processing conditions (Akhatou et al. 2017; Dong et al. 2025).

In contrast, CQA derivatives exhibited relatively stable SHAP value distributions across drying treatments, indicating that their contribution to classification was less affected by processing‐induced degradation and more influenced by intrinsic species‐level abundance differences. This stability supports their role as processing‐resilient markers that distinguish plant taxa rather than drying intensity. The complementary information provided by RF (global importance) and SHAP (local contribution and directionality) analyses thus enabled clear differentiation between processing‐sensitive markers (primarily flavonoid glycosides) and processing‐stable markers (predominantly caffeoylquinic acid derivatives).

**FIGURE 3:**
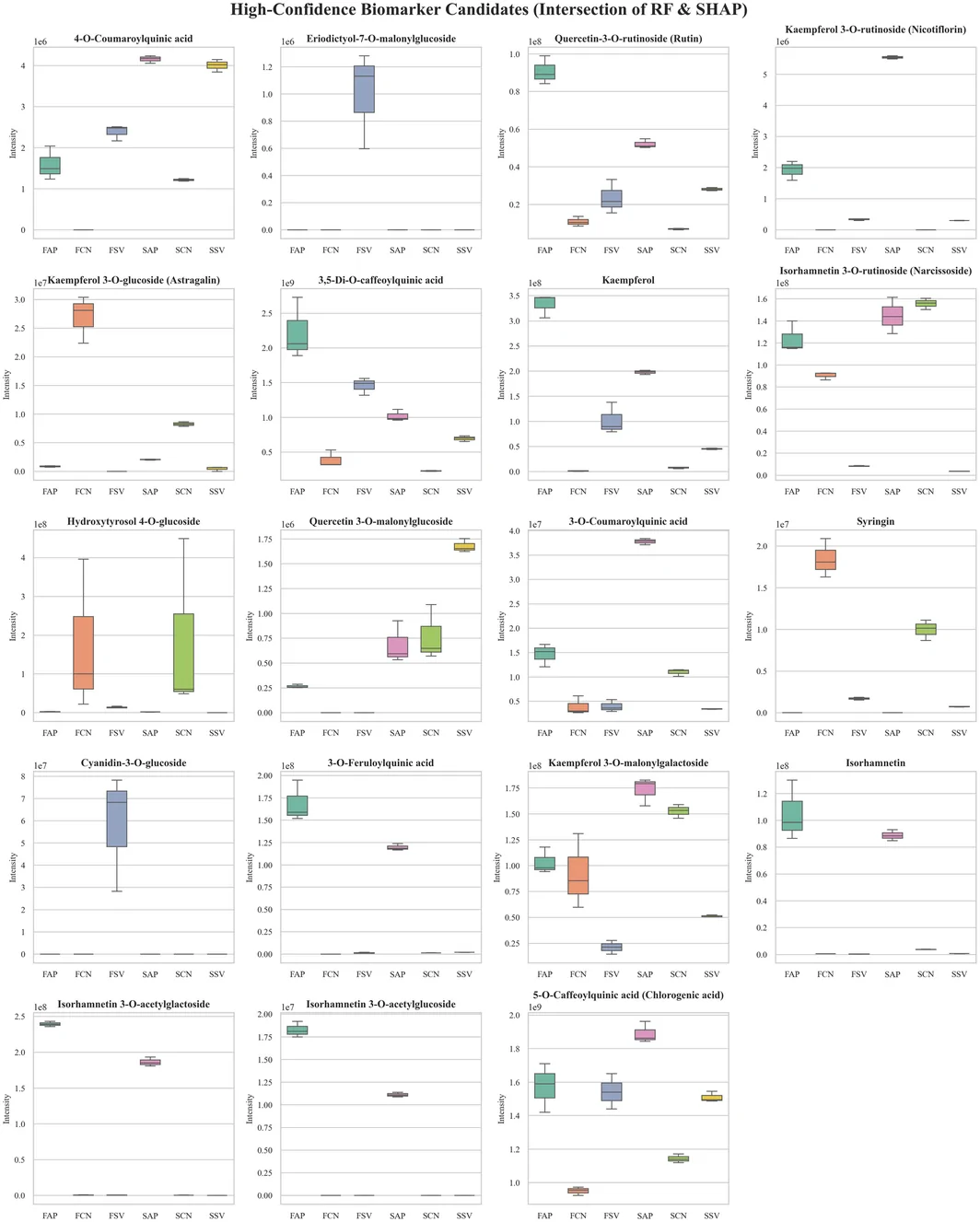
Boxplots of 19 shared top‐ranked metabolites identified by both RF and SHAP. Boxplots show the distribution of 19 metabolites jointly selected as important features by RF and SHAP.

To further refine marker selection, metabolites ranked among the top features based on both RF importance and absolute SHAP values were selected as high‐confidence candidates. Table 1 and Figure 3 present boxplots of 19 shared metabolites identified through this consensus approach, demonstrating consistent and statistically significant abundance patterns across species and drying conditions. These metabolites exhibited reduced intra‐group variability and clear inter‐group separation, supporting their robustness as discriminant features rather than artifacts of random variation or model overfitting. Using leave‐one‐out cross‐validation (LOOCV), the model achieved an accuracy of 1.00, a macro‐averaged F1‐score of 1.00, and a one‐versus‐rest ROC–AUC of 1.00 (Figure S4A), and the confusion matrix confirmed perfect separation among all six groups, with every sample correctly classified. Given the small sample size (*n* = 18) relative to the number of features (110), perfect LOOCV metrics may reflect optimistic performance estimates due to potential overfitting and should therefore be interpreted with caution. A label‐permutation test (1000 permutations) demonstrated that the observed accuracy and macro‐F1 scores (both 1.00) significantly exceeded the chance‐level null distribution (mean permuted accuracy = 0.104; empirical *p* = 0.001, Figure S4B). Repeated stratified 3‐fold cross‐validation (100 repeats) and repeated held‐out validation (100 random splits, one sample per class held out) also consistently yielded perfect classification performance. Nevertheless, as all validation procedures were performed on the same limited dataset, the model should be regarded as exploratory rather than externally validated.

**TABLE 1:** Key metabolite profiles in Asteraceae edible plants from Ulleungdo Island.

	Compound	Formula	ppm	Calc. MW	*m/z*	RT (min)	Adduct	Major fragment ions (*m/z*)
*Hydroxycinnamic acid*								
1	3*‐O‐*coumaroylquinic acid	C_16_ H_18_ O_8_	0.91	338.1005	337.0932	13.823	[M − H]^−1^	191.0556 (100), 93.0333 (29), 119.0491 (13), 163.0391 (10), 173.0446 (10), 87.0075 (8), 337.0941 (6), 85.0282 (6), 67.0175 (6), 111.0439 (5)
2	3*‐O‐*feruloylquinic acid	C_17_ H_20_ O_9_	−0.14	368.1107	367.1034	11.287	[M − H]^−1^	134.0363 (100), 193.0501 (70), 367.1044 (11), 117.0333 (9), 149.0598 (7), 191.0562 (4), 111.0438 (3), 155.0338 (3), 173.0448 (2), 93.0331 (2)
3	4*‐O‐*coumaroylquinic acid	C_16_ H_18_ O_8_	1.11	338.1005	337.0933	14.037	[M − H]^−1^	173.0447 (100), 93.0332 (39), 119.049 (24), 163.0391 (20), 111.044 (8), 337.0929 (7), 137.0234 (7), 67.0175 (6), 155.034 (5), 71.0125 (4)
4	5*‐O‐*caffeoylquinic acid (Chlorogenic acid)	C_16_ H_18_ O_9_	−0.16	354.095	353.0878	10.664	[M − H]^−1^	191.0556 (100), 85.0281 (8), 353.0855 (5), 220.7875 (4), 159.5122 (4), 83.3047 (3), 228.4181 (3), 213.0724 (3), 269.3395 (3), 50.0775 (3)
5	3,5‐Di*‐O‐*caffeoylquinic acid	C_25_ H_24_ O_12_	−0.17	516.1267	515.1194	20.974	[M − H]^−1^	191.0554 (100), 179.0343 (48), 135.0441 (48), 353.0888 (20), 515.1167 (6), 173.045 (6), 161.0236 (4), 93.0331 (3), 85.0281 (3), 407.123 (3)
*Flavonols*								
6	Isorhamnetin	C_16_ H_12_ O_7_	1.01	316.0586	315.0513	26.613	[M − H]^−1^	300.028 (100), 315.0516 (82), 151.0027 (24), 107.0126 (16), 271.0266 (7), 91.9577 (6), 148.0159 (5), 164.0105 (5), 283.0254 (5), 124.0153 (4)
7	Isorhamnetin 3*‐O‐*acetylgalactoside	C_24_ H_24_ O_13_	0.26	520.1218	519.1146	22.297	[M − H]^−1^	299.0196 (100), 314.0446 (100), 519.117 (76), 271.0248 (67), 315.0529 (58), 243.0293 (43), 300.0276 (29), 255.0303 (24), 262.9658 (21), 285.0414 (14)
8	Isorhamnetin 3*‐O‐*acetylglucoside	C_24_ H_24_ O_13_	1.08	520.1223	519.115	22.437	[M − H]^−1^	314.0439 (100), 519.1152 (95), 243.0299 (82), 271.0252 (69), 285.0408 (38), 299.0201 (31), 257.0459 (25), 270.0174 (16), 315.0523 (15), 300.0281 (10)
9	Isorhamnetin 3*‐O‐*rutinoside (Narcissoside)	C_28_ H_32_ O_16_	0.60	624.1694	623.1621	21.034	[M − H]^−1^	315.0515 (100), 623.1627 (69), 299.0201 (61), 300.0281 (48), 271.0251 (44), 314.044 (43), 243.0293 (29), 255.0298 (22), 285.0413 (10), 91.9648 (5)
10	Kaempferol	C_15_ H_10_ O_6_	0.56	286.0479	285.0406	26.157	[M − H]^−1^	285.0409 (100), 93.0334 (3), 185.0603 (3), 229.0499 (3), 187.0402 (2), 151.0026 (2), 107.0124 (2), 171.0442 (2), 239.0343 (2), 159.0441 (1)
11	Kaempferol 3*‐O‐*glucoside (Astragalin)	C_21_ H_20_ O_11_	0.51	448.1008	447.0935	21.074	[M − H]^−1^	255.0299 (100), 284.033 (83), 447.0941 (77), 227.0348 (74), 285.0411 (27), 256.0375 (6), 91.966 (4), 445.7786 (2), 443.458 (2), 444.4867 (2)
12	Kaempferol 3*‐O‐*malonylgalactoside	C_24_ H_22_ O_14_	1.10	534.1015	533.0943	22.157	[M − H]^−1^	284.033 (100), 255.03 (68), 489.1046 (40), 285.0407 (34), 227.0344 (22), 229.0497 (5), 257.0457 (4), 406.0903 (2), 431.6025 (2), 459.1559 (2)
13	Kaempferol 3*‐O‐*rutinoside (Nicotiflorin)	C_27_ H_30_ O_15_	1.19	594.1592	593.1519	21.114	[M − H]^−1^	285.0409 (100), 284.0331 (73), 593.152 (54), 255.0299 (53), 227.0347 (28), 229.0505 (17), 257.0454 (10), 256.0371 (6), 91.9656 (6), 151.0026 (5)
14	Quercetin 3*‐O‐*malonylglucoside	C_23_ H_22_ O_13_	1.46	506.1068	505.0995	21.358	[M − H]^−1^	300.0275 (100), 271.0251 (67), 505.0998 (41), 255.0297 (30), 301.0351 (18), 151.0028 (13), 91.965 (12), 238.4418 (6), 98.7616 (5), 61.0849 (5)
15	Quercetin 3*‐O‐*rutinoside (Rutin)	C_27_ H_30_ O_16_	0.66	610.1538	609.1465	19.461	[M − H]^−1^	300.0279 (100), 609.1466 (60), 271.0252 (50), 301.0356 (30), 255.0297 (24), 151.0027 (10), 178.9975 (7), 243.03 (6), 254.0208 (4), 163.0035 (3)
*Flavanones*								
16	Eriodictyol‐7*‐O‐*malonylglucoside	C_24_ H_24_ O_14_	1.58	536.1175	535.1102	15.543	[M − H]^−1^	269.0459 (100), 225.0555 (23), 197.0603 (22), 493.1354 (18), 350.0928 (18), 547.508 (17), 76.1872 (15), 98.5952 (15), 73.8299 (15), 91.9689 (15)
*Anthocyanins*								
17	Cyanidin‐3*‐O‐*glucoside	C_21_ H_20_ O_11_	−0.11	448.1005	449.1078	12.346	[M + H]^+1^	287.0549 (100), 449.1074 (23), 449.0477 (2), 449.1684 (2), 357.1077 (1), 147.8942 (1), 148.5419 (1), 116.8668 (1), 98.5976 (1), 74.9836 (1)
*Phenylpropanoid*								
18	Syringin	C_17_ H_24_ O_9_	−0.95	372.1417	373.1493	11.392	[M + H]^+1^	395.1313 (100), 232.0708 (10), 233.0785 (6), 185.0427 (3), 364.1161 (2), 396.1318 (2), 148.9835 (1), 229.2643 (1), 218.1463 (1), 105.5037 (1)
*Phenylethanoid*								
19	Hydroxytyrosol 4*‐O‐*glucoside	C_14_ H_20_ O_8_	0.75	316.1161	315.1088	5.712	[M − H]^−1^	315.1091 (100), 135.0441 (97), 71.0124 (67), 89.0232 (58), 153.0548 (47), 123.044 (41), 101.023 (39), 119.0336 (21), 113.0231 (18), 92.4184 (2)

**FIGURE 4:**
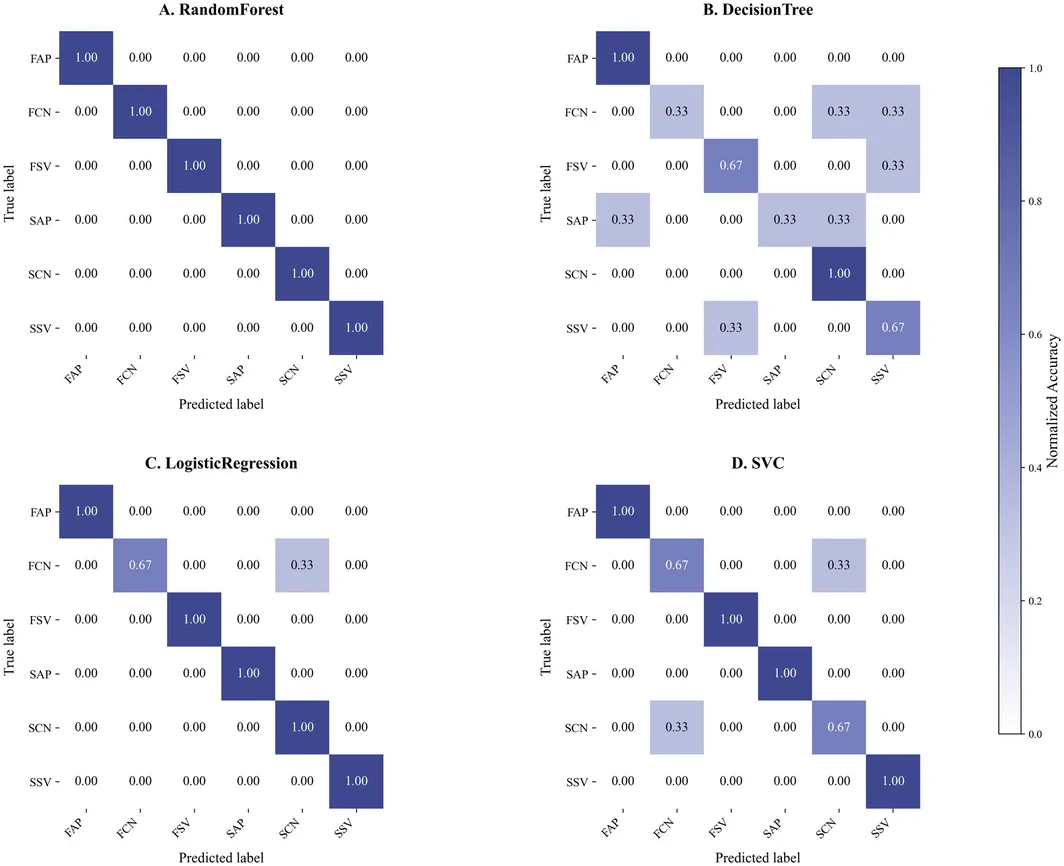
Confusion matrices of four classification algorithms: (A) Random Forest, (B) Decision Tree, (C) Logistic Regression, and (D) Support Vector Classifier. Model validation is presented in Figure S4.

To confirm that the observed group separation was not specific to a single algorithm, three additional classifiers were compared with RF. Random Forest achieved complete separation of all six groups, whereas Decision Tree, Logistic Regression, and Support Vector Classifier showed misclassification, most frequently between FCN and SCN (Figure 4). This comparison supports the use of Random Forest as the base model for SHAP interpretation.

Overall, the integrated RF–SHAP analysis provided a transparent and statistically robust framework for identifying candidate marker compounds associated with both taxonomic identity and post‐harvest drying conditions. The convergence of RF feature importance, SHAP‐based interpretability, and model validation supports the use of CQA derivatives as stable chemotaxonomic markers and glycosylated flavonoids as sensitive indicators of processing intensity. This differential behavior can be partly explained by their structural characteristics. Flavonoid O‐glycosides contain glycosidic linkages that are more susceptible to hydrolysis and subsequent oxidation during thermal and photo‐oxidative processing, resulting in greater abundance changes than those observed for the relatively more stable caffeoylquinic acid derivatives (Wianowska and Gil 2019; Xiao 2022). Consequently, glycosylated flavonoids exhibited stronger discriminatory power in the RF–SHAP models and served as highly informative markers of processing intensity. These findings lay the foundation for targeted quantification and the development of metabolite‐based quality control strategies for traditionally processed wild vegetables from Ulleungdo.

### 3.3 Quantification of Key Metabolites

**FIGURE 5:**
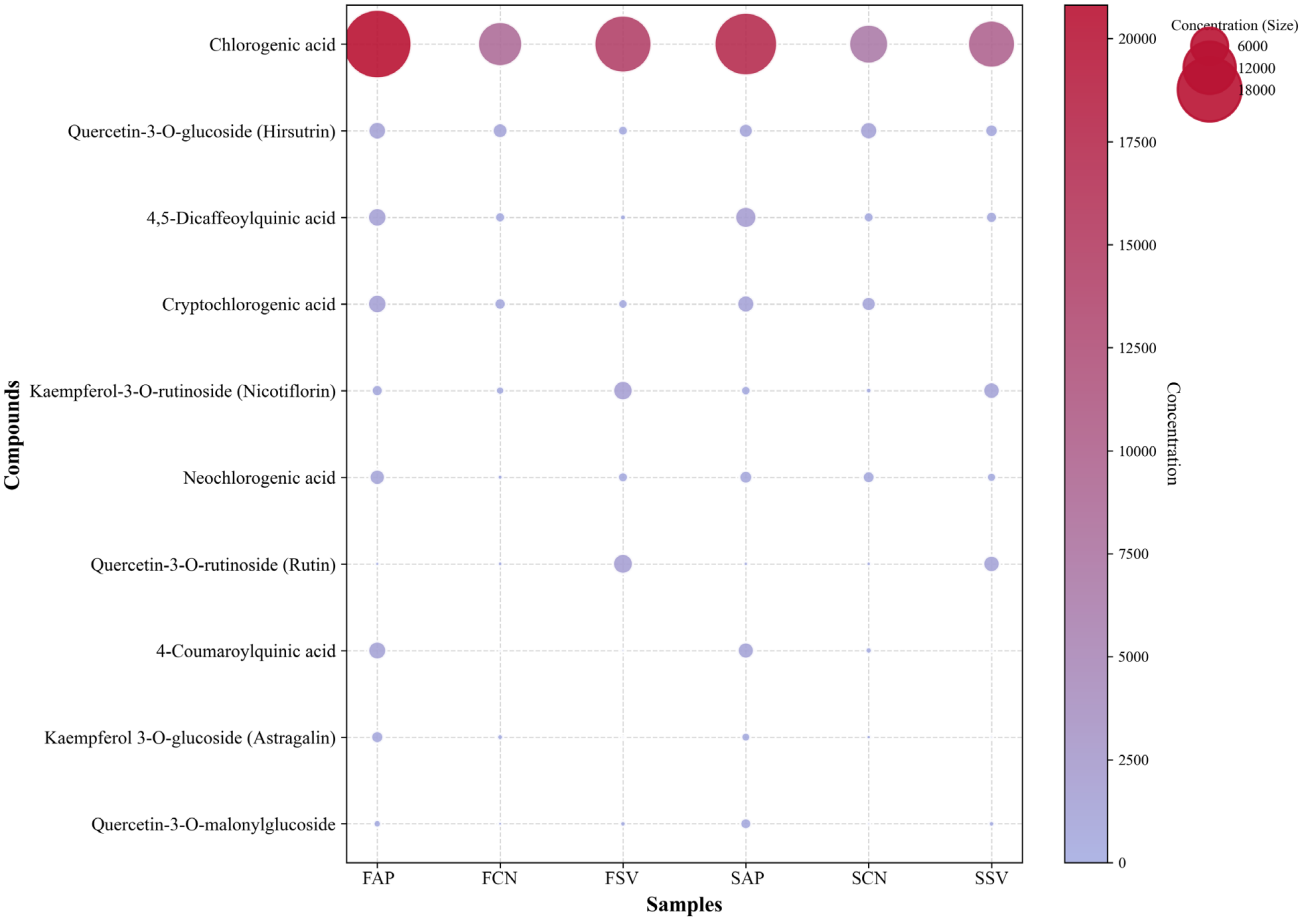
Quantitative profiles of the top 10 phenolic metabolites visualization of the 10 most abundant phenolic compounds quantified in the three Asteraceae species under freeze‐dried and sun‐dried conditions.

A quantitative evaluation of the major phenolic metabolites provided a detailed understanding of how species identity and processing method jointly influence the phytochemical composition of the three Asteraceae edible plants examined in this study (FAP, FCN, FSV for freeze‐dried samples; SAP, SCN, SSV for sun‐dried samples) (Table 2, Figure 5). Across all samples, the 10 most abundant compounds were predominantly CQA derivatives and glycosylated flavonoids, reflecting the characteristic phenolic profiles of *A. pseudoglehnii*, *C. nipponicum* , and *S. virgaurea*. However, the relative abundance of these subclasses showed clear divergence depending on whether samples were freeze‐dried or naturally sun‐dried, revealing processing‐specific metabolic responses that closely align with the RF–SHAP‐derived biomarker profiles.

**TABLE 2:** Quantification of major phenolic metabolites in Asteraceae edible plants from Ulleungdo Island.

#	Compounds (mg/kg dm)	FAP	FCN	FSV	SAP	SCN	SSV	*F*‐value
*Hydroxycinnamic acid*								
1	3*‐O‐*coumaroylquinic acid	1073.55 ± 135.23	125.24 ± 105.37	469.03 ± 74.03	759.15 ± 136.33	649.50 ± 11.45	396.60 ± 20.26	35.55***
2	3*‐O‐*feruloylquinic acid	20.43 ± 1.75	11.85 ± 2.55	64.68 ± 9.99	33.54 ± 2.17	9.24 ± 0.25	55.74 ± 1.71	82.72***
3	3,5‐Di*‐O‐*caffeoylquinic acid	1552.48 ± 311.83	473.10 ± 242.46	172.30 ± 39.76	2031.75 ± 583.57	461.26 ± 25.78	574.28 ± 46.43	19.50***
4	4*‐O‐*coumaroylquinic acid	1462.70 ± 78.10	11.67 ± 2.57	44.60 ± 9.08	1185.86 ± 234.82	198.41 ± 5.61	34.43 ± 1.34	126.14***
5	5*‐O‐*caffeoylquinic acid	20,812.14 ± 2455.17	8698.56 ± 3623.54	14,465.69 ± 1909.06	17,394.99 ± 3364.25	6777.31 ± 187.56	9829.19 ± 663.03	15.59***
6	5*‐O‐*feruloylquinic acid	145.70 ± 3.88	nd	2.02 ± 0.26	230.02 ± 30.68	1.81 ± 0.13	2.61 ± 0.10	187.54***
7	Cryptochlorogenic acid	1547.00 ± 149.16	594.99 ± 130.03	417.39 ± 64.92	1322.37 ± 244.13	927.46 ± 6.49	nd	58.56***
8	Ferulic acid	160.64 ± 27.76	nd	nd	264.23 ± 35.63	nd	nd	115.65***
*Flavones*								
9	Apigenin	0.01 ± 0.01	1.86 ± 0.17	0.01 ± 0.00	nd	14.75 ± 0.20	0.02 ± 0.00	9002.70***
10	Apigenin 6,8‐C‐diglucoside (Vicenin 2)	nd	14.94 ± 9.55	nd	nd	99.66 ± 2.44	nd	295.35***
11	Apigenin 6‐C‐glucoside (Isovitexin)	nd	0.28 ± 0.05	nd	nd	9.52 ± 0.35	nd	2141.77***
12	Chrysoeriol	1.02 ± 0.27	nd	nd	1.25 ± 0.04	1.28 ± 0.17	nd	73.80***
13	Diosmetin	nd	0.82 ± 0.38	nd	nd	17.95 ± 0.29	nd	4182.91***
14	Luteolin	nd	nd	nd	nd	0.66 ± 0.04	nd	891.71***
15	Luteolin 7*‐O‐*glucoside (Cynaroside)	0.34 ± 0.06	0.72 ± 0.51	0.12 ± 0.02	0.15 ± 0.00	1.45 ± 0.07	0.14 ± 0.02	17.95***
16	Scutellarein	nd	nd	nd	nd	1.24 ± 0.15	nd	193.95***
*Flavonols*								
17	Isorhamnetin	17.74 ± 1.37	0.03 ± 0.01	0.05 ± 0.01	9.34 ± 2.43	0.08 ± 0.00	0.17 ± 0.02	127.85***
18	Isorhamnetin 3*‐O‐*rutinoside (Narcissoside)	256.19 ± 28.24	93.23 ± 3.92	13.03 ± 2.90	240.30 ± 29.96	118.52 ± 1.31	3.81 ± 0.40	122.77***
19	Kaempferide	nd	nd	0.02 ± 0.00	nd	0.06 ± 0.04	nd	8.30**
20	Kaempferol	65.40 ± 5.18	0.80 ± 0.11	31.76 ± 9.99	108.36 ± 4.61	2.17 ± 0.06	14.30 ± 0.61	217.64***
21	Kaempferol 3*‐O‐*glucoside (Astragalin)	681.96 ± 87.52	171.45 ± 105.47	20.28 ± 5.23	368.18 ± 77.55	105.00 ± 1.54	38.89 ± 1.58	46.82***
22	Kaempferol 3*‐O‐*rhamnoside (Afzelin)	nd	nd	nd	nd	0.12 ± 0.02	nd	144.54***
23	Kaempferol‐3*‐O‐*rutinoside (Nicotiflorin)	575.09 ± 70.81	330.44 ± 164.76	1714.26 ± 192.31	426.45 ± 66.72	160.98 ± 0.67	1242.80 ± 67.63	84.30***
24	Kaempferol‐3‐sambubioside	11.09 ± 1.37	nd	nd	16.70 ± 2.48	nd	nd	122.66***
25	Quercetin	4.93 ± 0.63	0.17 ± 0.02	7.15 ± 2.23	6.18 ± 0.89	1.12 ± 0.04	13.25 ± 0.04	65.08***
26	Quercetin‐3*‐O‐*glucoside (Hirsutrin)	1406.28 ± 185.97	996.46 ± 191.75	453.06 ± 236.99	901.90 ± 152.64	1347.89 ± 26.41	726.88 ± 36.30	15.66***
27	Quercetin‐3*‐O‐*malonylglucoside	259.48 ± 36.42	79.62 ± 13.13	153.81 ± 70.93	556.11 ± 86.42	42.35 ± 2.26	155.85 ± 17.43	43.63***
28	Quercetin‐3*‐O‐*rhamnoside (Quercitrin)	nd	nd	nd	nd	0.44 ± 0.04	0.18 ± 0.05	159.91***
29	Quercetin‐3*‐O‐*rutinoside (Rutin)	90.99 ± 9.35	110.63 ± 22.99	1783.21 ± 390.42	101.48 ± 20.22	104.81 ± 3.46	1232.73 ± 64.54	63.66***
*Flavanones*								
30	Eriodictyol	nd	0.06 ± 0.02	nd	nd	0.32 ± 0.11	0.05 ± 0.01	22.94***
31	Naringenin	0.21 ± 0.02	0.73 ± 0.22	0.17 ± 0.08	0.03 ± 0.01	2.22 ± 0.03	0.19 ± 0.01	226.41***
*Flavanols*								
32	Catechin	nd	0.27 ± 0.44	nd	nd	nd	nd	1.11
33	Epicatechin	nd	2.18 ± 1.82	nd	nd	nd	nd	4.30*

*Note:* Mean ± SD concentrations (*n* = 3) of key phenolic compounds in freeze‐dried samples FAP (*Aster pseudoglehnii*), FCN (*Cirsium nipponicum*), FSV (*Solidago virgaurea*) and sun‐dried samples SAP (*Aster pseudoglehnii*), SCN (*Cirsium nipponicum*), SSV (*Solidago virgaurea*). *F*‐values were obtained by one‐way ANOVA.

Abbreviations: dm, dry matter; nd, not detected.

* *p* < 0.05.

** *p* < 0.01.

*** *p* < 0.001.

The most notable trend was the overall stability of CQA derivatives during sun‐drying. Chlorogenic acid, 3,5‐diCQA, 4,5‐diCQA, and other mono‐ and di‐acyl CQAs retained relatively high concentrations in SAP, SCN, and SSV compared to their freeze‐dried counterparts. However, this apparent retention may partly reflect concentration effects associated with moisture loss during drying rather than solely chemical stability. In several cases, the concentration ratios (sun‐dried/freeze‐dried) approached equivalence, indicating minimal degradation under the slow, low‐temperature drying conditions characteristic of traditional sun‐drying. This observation supports previous stability studies demonstrating that CQA esters undergo limited thermal decomposition at moderate temperatures and are far less susceptible to hydrolysis than other phenolic subclasses (Xue et al. 2016). Furthermore, the preferential retention of di‐CQA isomers may be attributed to their higher structural rigidity and lower reactivity under oxidative conditions, as previously reported in model system degradation assays.

In contrast, glycosylated flavonoids—particularly rutin, quercetin 3‐*O*‐glucoside, kaempferol 3‐*O*‐glucoside, and their malonylated derivatives—exhibited significant decreases in sun‐dried samples compared to freeze‐dried samples. These reductions were consistent across the three species but varied in magnitude, with the most rapid loss observed in *S. virgaurea* samples (FSV → SSV). Because blanching was performed only prior to sun‐drying, the observed decreases should be interpreted as reflecting the combined effects of blanching and the subsequent drying process rather than sun‐drying alone. Accordingly, the relative contributions of blanching and drying cannot be distinguished in the present experimental design. Nevertheless, these findings align with previous reports indicating that O‐glycosidic linkages in flavonoids are susceptible to enzymatic deglycosylation, photo‐oxidation, and processing‐related degradation during postharvest dehydration (Ianni et al. 2022; Tang et al. 2025). In particular, prolonged exposure to sunlight during drying may have contributed to photo‐oxidative degradation of flavonoid aglycones, although the influence of the preceding blanching step cannot be excluded. The observed species‐specific sensitivity may reflect inherent differences in vacuolar organization, glycosyltransferase activity, or endogenous polyphenol oxidase levels, all of which influence postharvest metabolite stability.

Importantly, the quantitative patterns strongly align with the machine learning results obtained from the RF–SHAP analysis. Compounds identified as high‐importance biomarkers for distinguishing processing conditions—particularly CQA—demonstrated measurable stability across drying treatments, reinforcing their suitability as robust chemical markers. Conversely, the consistent reduction of glycosylated flavonoids in sun‐dried samples supports the SHAP‐derived indication that these compounds drive the separation between freeze‐dried and sun‐dried groups due to their sensitivity to processing. This convergence of untargeted chemometric and targeted quantitative evidence enhances the interpretive validity of the biomarkers identified in this study.

Species‐level comparisons further revealed distinct phenolic allocation strategies. *A. pseudoglehnii* samples (FAP/SAP) exhibited the most balanced distribution between CQA derivatives and flavonoid glycosides, suggesting dual contributions from both the hydroxycinnamate and flavonoid biosynthetic pathways. *C. nipponicum* (FCN/SCN) showed the highest baseline abundance of CQAs, consistent with previous studies identifying *Cirsium* species as CQA‐rich wild edible greens. The *S. virgaurea* (FSV/SSV) exhibited the highest flavonoid content among the freeze‐dried samples but also showed the greatest relative reduction following the combined blanching and sun‐drying treatment. This finding suggests that its flavonoid pool is more susceptible to processing‐related degradation, particularly photo‐oxidation and prolonged enzymatic activity during the extended drying period. The dominant phenolic subclass present in each species may influence the overall stability of the metabolome during drying. Species enriched in CQAs retained relatively stable phenolic profiles, whereas species containing higher proportions of flavonoid glycosides exhibited greater compositional changes following traditional processing. This observation suggests that the initial biochemical composition partially determines the susceptibility of individual metabolites to oxidation and hydrolysis during post‐harvest treatment. From a functional perspective, the selective preservation of CQA derivatives during sun‐drying suggests that sun‐dried shoots may retain substantial antioxidant potential, given the well‐established radical‐scavenging and anti‐inflammatory properties of CQAs (Alcázar Magaña et al. 2021). However, the reduced levels of flavonoid glycosides—which also contribute significantly to antioxidant and anti‐inflammatory activities—indicate a potential shift in the bioactive profile of sun‐dried materials. This differential retention may also reflect interactions among phenolic constituents within the same extract. As major components of the antioxidant system, abundant phenolic compounds may contribute to the overall redox environment by scavenging reactive oxygen species, thereby reducing oxidative degradation of more labile metabolites (Shahidi and Ambigaipalan 2015). Conversely, depletion of these predominant antioxidant pools during prolonged drying could increase the susceptibility of oxidation‐sensitive compounds, including flavonoid glycosides, to further degradation. Although these interactions were not directly investigated in the present study, they provide a plausible explanation for the species‐dependent metabolomic responses observed following different drying treatments. For food and nutraceutical applications, these findings underscore the importance of considering not only total phenolic content but also subclass‐specific retention patterns when selecting drying methods. Freeze‐drying remains preferable for preserving the full spectrum of photo‐ and enzymatically labile flavonoids, whereas sun‐drying is suitable when retention of CQA‐dominant profiles is desired or when traditional processing is integral to product identity.

Overall, the quantitative analysis reveals that processing conditions play a crucial role in modulating metabolite stability across Asteraceae species. The consistent alignment between targeted quantification and machine learning‐derived biomarker importance further demonstrates that an integrated chemometric–biochemical approach provides a powerful framework for elucidating processing‐induced metabolic changes in wild edible plants.

## 4 Conclusion

This study provides an integrative metabolomic and machine learning analysis of three Asteraceae wild vegetables from Ulleungdo, demonstrating that species identity is the primary determinant of phytochemical composition, while drying methods induce predictable, subclass‐specific metabolic shifts. Untargeted UHPLC–HRMS profiling and hierarchical clustering revealed distinct chemotypes for *C. nipponicum* , *S. virgaurea*, and *A. pseudoglehnii*, with traditional processing (blanching followed by sun‐drying) consistently reducing processing‐labile flavonol glycosides and preserving or enriching CQA that remained comparatively stable under these combined conditions. RF–SHAP analysis further identified discriminant markers capturing both species‐specific and processing‐dependent metabolic features. Targeted quantification validated these patterns, showing that *C. nipponicum* is enriched in di‐CQAs with strong antioxidant potential, *S. virgaurea* retains relatively stable C‐glycosyl flavones linked to immune‐modulating activity, and *A. pseudoglehnii* offers bioactive apigenin and chrysoeriol derivatives best preserved by freeze‐drying. Collectively, these findings highlight the complementary influences of taxonomy and post‐harvest processing on the phenolic profiles of Ulleungdo Asteraceae, identify candidate markers for quality assessment, and provide a biochemical basis for optimizing processing strategies to enhance the nutritional and functional value of these regionally important food resources.

## Author Contributions

**Doo‐Hee Lee:** writing – review and editing, methodology, validation. **Nami Joo:** writing – review and editing, resources, project administration. **Ju Hong Park:** funding acquisition, resources, project administration. **In Young Lee:** conceptualization, investigation, methodology, software, data curation, formal analysis, supervision, visualization, project administration, resources, writing – original draft, writing – review and editing, validation.

## Funding

This work was supported by a grant of the FoodTech RnD Center Development and Support Program through the Gyeongbuk Technopark (GBTP) funded by GYEONGSANGBUK‐DO and Pohang city (GBTP2023129001), by a grant of the High Value‐added Food Technology Development Program through the Korea Institute of Planning and Evaluation for Technology in Food, Agriculture and Forestry (IPET) funded by the Ministry of Agriculture, Food and Rural Affairs (MAFRA, RS‐2024‐00403998 and RS‐2024‐00403987), and by a grant of the Korea Institute of Planning and Evaluation for Technology in Food, Agriculture and Forestry (IPET) through Agriculture and Food Convergence Technologies Program for Research Manpower development Program, funded by the Ministry of Agriculture, Food and Rural Affairs (MAFRA, RS‐2024‐00402136).

## Ethics Statement

The authors have nothing to report.

## Conflicts of Interest

The authors declare no conflicts of interest.

## Supporting information


**Table S1:** Parameters of compound discoverer workflow.
**Table S2:** SRM parameters of phenolic compounds.
**Table S3:** Method validation parameters of phenolic compounds.
**Table S4:** Metabolite profiles in Asteraceae edible plants from Ulleungdo Island.
**Figure S1:** Calibration curves of phenolic compounds used for targeted quantification.
**Figure S2:** Negative mode of total ion chromatogram (A: freeze‐dried *Aster pseudoglehnii* [FAP], B: freeze‐dried *Cirsium nipponicum* [FCN], C: freeze‐dried *Solidago virgaurea* [FSV], D: sun‐dried *Aster pseudoglehnii* [SAP], E: sun‐dried *Cirsium nipponicum* [SCN], F: sun‐dried *Solidago virgaurea* [SSV]).
**Figure S3:** Positive mode of total ion chromatogram (A: freeze‐dried *Aster pseudoglehnii* [FAP], B: freeze‐dried *Cirsium nipponicum* [FCN], C: freeze‐dried *Solidago virgaurea* [FSV], D: sun‐dried *Aster pseudoglehnii* [SAP], E: sun‐dried *Cirsium nipponicum* [SCN], F: sun‐dried *Solidago virgaurea* [SSV]).
**Figure S4:** Internal validation of the Random Forest classification model. (A) Confusion matrix obtained using leave‐one‐out cross‐validation (LOOCV). (B) Distribution of classification accuracy from 1000 label permutations.

## Data Availability

The data that support the findings of this study are available from the corresponding author upon reasonable request.
