# Supplementary material for: Integrated Metabolomics and Machine Learning Reveal Drying‐Dependent Phenolic Markers in Asteraceae Edible Plants From Ulleungdo Island
Source: Food Sci Nutr. 2026 Sep 2;14(9):e72297. doi: 10.1002/fsn3.72297 (PMC13536481; doi:10.1002/fsn3.72297)
Supplement: Supplementary file 1 [file FSN3-14-e72297-s001.docx]

Table S1. Parameters of Compound Discoverer workflow.

| Workflow Tree | Workflow Parameters | Workflow Information |
| --- | --- | --- |
| Input files | - | Q-Exactrive raw data (.Raw) |
| Select Spectra | Spectrum Properties Filter | Lower RT limit: 0  Upper RT limit: 0 |
|  | Scan Event Filters | Polarity Mode: Any |
| Align Retention Times | General Settings | Reference File: None |
| Detect Compounds | General Settings | Mass Tolerance: 3 ppm  Min, Peak Intensity: 100,000  Use Most Intense Isotope Only: True |
|  | Peak Detection | Chromatographic S/N Threshold: 5  Remove Baseline: True |
|  | Compound Detection | Ions: [2M + H]+1; [2M + Na]+1; [M + 2H]+2 [M + H]+1; [M + H – H2O]+1; [M + Na]+1; [M -e]+1; [M + NH4]+1; [M –NH3+H]+1 ; [M - 2H]-2; [M -H]-1; [M + FA - H]-1; [2M - H]-1; |
| Group Compounds | - | Mass Tolerance: 3 ppm  RT Tolerance [min]: 0.3  Preferred Ions: [M - H] +1; [M- H] -1  Area Integration: Most Common Ion |
| Predict Compositions | Prediction Settings | Mass Tolerance: 3 ppm  Min. Element Counts: C. H, O  Max. Element Counts: C100 H200 N50 O100 P50 S50  Min. RDBE: 0  Max. RDBE: 40  Min. H/C: 0.1  Max. H/C: 3.5  Max.# Candidates: 5 |
|  | Pattern Matching | Intensity Tolerance [%]: 50  Intensity Threshold [%]: 0.1  S/N Threshold: 5  Use Dynamic Recalibration: True |
|  | Fragments Matching | Use Fragments Matching: True  Mass Tolerance: 5 ppm  S/N Threshold: 10 |
| Search mzCloud | General Settings | Compound Classes: All  Library: Autoprocessed, Reference  Search MSn Tree: False |
|  | DDA Search | Identity Search: HighChem HighRes  Match Activation Type: True  Match Activation Energy: Match with Tolerance  Activation Energy: 20  Apply Intensity Threadhold: True |
| Search mzVault | Search Settings | MzVault Library: Bamba lab 34 lipid mediators library.db; Bamba lab 598 polar metabolites library.db; LipidBlast-VS68-Neg.db; LipidBlast-VS68-Pos.db; NIST_2020_MSMS_HR.db; Mona_MSMS_Negative.db; Mona_MSMS_Positive.db; MoNA-export-LipidBlast_2022.db; Inhouse Library_Negative.db; Inhouse Library_Positive.db  Precursor Mass Tolerance: 5 ppm  Search Algorithm: HighChem HighRes  Match Factor Threshold: 50 |
| Search ChemSpider | Search Settings | Databases: Food and Agriculture Organization of the United Nations; FooDB; KEGG; NIST; NIST Spectra; PlantCyc  Search Mode: By Formula or Mass  Mass Tolerance: 5 ppm  Max.# of results per compound: 50  Max.# of Predicted Composition: 5 |
| Map to Metabolika Pathways | Search Settings | Metabolika pathways: All  Search Mode: By Formula or Mass |
|  | By Mass Search Settings | Mass Tolerance: 5 ppm |
|  | By Formula Search Settings | Max. # of Predicted Compositions to be searched per Compound: 3 |
|  | Display Settings | Max. # of Pathways in ‘Pathways’ column: 20 |
| Assign Compound Annotations | General Settings | Mass Tolerance: 5 ppm |
|  | Data Sources | Data source #1: Predicted Compositions  Data source #2: mzCloud Search  Data source #3: mzVault Search  Data source #4: ChemSpider Search  Data source #5: Metabolika Search |
| Fill Gaps | General Settings | Mass Tolerance: 2 ppm  S/N Threshold: 5 |

Table S2. SRM parameters of phenolic compounds

| # | Compound | Adduct | Precursor Ion (m/z) | Quantifier Ion (m/z) [CE (V)] | Qualifier Ion 1 (m/z) [CE (V)] | Qualifier Ion 2 (m/z) [CE (V)] |
| --- | --- | --- | --- | --- | --- | --- |
|  | *Hydroxycinnamic acid* |  |  |  |  |  |
| 1 | 3*-O-*coumaroylquinic acid | [M-H]- | 337.0 | 162.9 [14.1] | 216.9 [10.6] | 218.9 [18.1] |
| 2 | 3*-O-*feruloylquinic acid | [M-H]- | 367.1 | 191.1 [14.5] | 173.1 [17.4] | 193.1 [22.4] |
| 3 | 3,5-Di*-O-*caffeoylquinic acid | [M-H]- | 515.0 | 353.1 [18.2] | 173.1 [29.1] | 179.1 [29.4] |
| 4 | 4*-O-*coumaroylquinic acid | [M-H]- | 353.1 | 191.1 [18.9] | 135.1 [31.0] | 179.1 [18.0] |
| 5 | 5*-O-*feruloylquinic acid | [M-H]- | 367.1 | 193.1 [19.8] | 173.1 [18.1] | 191.1 [15.8] |
| 6 | 5*-O-*caffeoylquinic acid | [M-H]- | 353.1 | 191.1 [16.4] | 85.1 [40.1] | 179.1 [15.0] |
| 7 | Cryptochlorogenic acid | [M-H]- | 353.1 | 173.1 [16.1] | 179.1 [15.4] | 191.1 [22.8] |
| 8 | Ferulic acid | [M-H]- | 192.9 | 134.0 [14.4] | 149.0 [16.5] | 137.1 [15.7] |
|  | *Flavones* |  |  |  |  |  |
| 9 | Apigenin | [M-H]- | 269.1 | 117.1 [35.0] | 149.1 [24.3] | 151. 1 [24.5] |
| 10 | Apigenin 6,8-C-diglucoside (Vicenin 2) | [M-H]- | 593.2 | 353.1 [36.2] | 383.1 [35.8] | 473.2 [27.6] |
| 11 | Apigenin 6-C-glucoside (Isovitexin) | [M+H]+ | 433.1 | 283.1 [25.9] | 337.1 [18.5] | 367.1 [15.4] |
| 12 | Chrysoeriol | [M-H]- | 299.1 | 284.1 [21.9] | 227.1 [35.3] | 256.1 [28.2] |
| 13 | Diosmetin | [M-H]- | 299.1 | 284.0 [22.3] | 227.0 [37.3] | 256.0 [30.2] |
| 14 | Luteolin | [M+H]+ | 287.1 | 153.0 [32.7] | 135. 1 [32.5] | 165.0 [27.7] |
| 15 | Luteolin 7-O-glucoside (Cynaroside) | [M-H]- | 447.1 | 285.1 [25.8] | 300.1 [25.1] | 301.1 [21.2] |
| 16 | Scutellarein | [M-H]- | 285.1 | 117.0 [39.3] | 137.0 [30.9] | 239.0 [24.0] |
|  | *Flavonols* |  |  |  |  |  |
| 17 | Isorhamnetin | [M-H]- | 315.1 | 300.2 [21.6] | 151.1 [28.9] | 271.1 [29.9*] |
| 18 | Isorhamnetin 3-O-rutinoside (Narcissoside) | [M-H]- | 623.3 | 315.1 [29.0] | 299.1 [26.8] | 300.2 [44.1] |
| 19 | Kaempferide | [M-H]- | 299.1 | 284.0 [21.9] | 151.0 [29.9] | 164.0 [31.4] |
| 20 | Kaempferol | [M+H]+ | 287.2 | 153.0 [33.6] | 121.1 [32.7] | 165.1 [28.9] |
| 21 | Kaempferol 3-O-glucoside (Astragalin) | [M-H]- | 447.1 | 255.1 [39.5] | 227.1 [46.3] | 284.1 [26.5] |
| 22 | Kaempferol 3-O-rhamnoside (Afzelin) | [M-H]- | 431.1 | 285.1 [19.8] | 255.1 [26.5] | 284.1 [25.6] |
| 23 | Kaempferol 3-O-rutinoside (Nicotiflorin) | [M-H]- | 593.2 | 285.1 [30.5] | 255.1 [53.3] | 284.1 [35.5] |
| 24 | Kaempferol 3-O-sambubioside | [M-H]- | 579.2 | 284.1 [31.6] | 227.1 [54.7] | 255.1 [51.8] |
| 25 | Quercetin | [M-H]- | 301.0 | 151.1 [21.0] | 121.1 [25.6] | 179.1 [17.6] |
| 26 | Quercetin 3-O-glucoside (Hirsutrin) | [M-H]- | 463.0 | 300.1 [26.7] | 271.1 [42.5] | 301.1 [21.9] |
| 27 | Quercetin 3-O-malonylglucoside | [M+H]+ | 551.1 | 303.1 [17.4] | 301.0 [20.6] | 85.1 [26.8] |
| 28 | Quercetin 3-O-rhamnoside (Quercitrin) | [M-H]- | 447.1 | 300.1 [25.1] | 271.1 [42.5] | 301.1 [21.9] |
| 29 | Quercetin 3-O-rutinoside (Rutin) | [M-H]- | 609.1 | 300.1 [25.1] | 271.1 [42.5] | 301.1 [21.9] |
|  | *Flavanones* |  |  |  |  |  |
| 30 | Eriodictyol | [M-H]- | 287.0 | 151.0 [14.8] | 107.0 [24.9] | 135.1 [25.9] |
| 31 | Naringenin | [M-H]- | 271.1 | 151.1 [17.6] | 119.1 [26.1] | 177.1 [16.8] |
|  | *Flavanols* |  |  |  |  |  |
| 32 | Catechin | [M-H]- | 289.0 | 245.1 [14.5] | 203.1 [19.2] | 205.0 [16.8] |
| 33 | Epicatechin | [M-H]- | 289.0 | 245.1 [14.6] | 203.0 [18.9] | 205.0 [17.0] |

Table S3. Method validation parameters of phenolic compounds

| # | Compound | RT  (min) | Linear range  (ng/mL) | R^2^ | LOD  (ng/g) | LOQ  (ng/g) | Accuracy  (%) | Precision  (%) |
| --- | --- | --- | --- | --- | --- | --- | --- | --- |
|  | *Hydroxycinnamic acid* |  |  |  |  |  |  |  |
| 1 | 3-O-Coumaroylquinic acid | 3.71 | 1-1000 | 0.9999 | 0.344 | 1.043 | 82.96 | 1.90 |
| 2 | 3*-O-*feruloylquinic acid | 4.81 | 1-500 | 0.9999 | 0.354 | 1.071 | 84.26 | 3.28 |
| 3 | 3,5-Di-O-caffeoylquinic acid | 5.78 | 1-500 | 0.9998 | 0.539 | 1.634 | 87.41 | 2.27 |
| 4 | 4-O-coumaroylquinic acid | 4.07 | 1-1000 | 0.9998 | 0.478 | 1.447 | 84.34 | 1.43 |
| 5 | 5-O-caffeoylquinic acid | 4.14 | 5-1000 | 0.9999 | 0.457 | 1.384 | 82.83 | 2.92 |
| 6 | 5-O-feruloylquinic acid | 4.25 | 1-1000 | 0.9998 | 0.499 | 1.512 | 86.15 | 2.78 |
| 7 | Cryptochlorogenic acid | 4.20 | 5-1000 | 0.9998 | 0.580 | 1.756 | 82.43 | 1.20 |
| 8 | Ferulic acid | 4.25 | 2-200 | 0.9999 | 0.617 | 1.868 | 85.12 | 3.43 |
|  | *Flavones* |  |  |  |  |  |  |  |
| 9 | Apigenin | 7.71 | 1-100 | 0.9996 | 0.532 | 1.610 | 86.65 | 2.08 |
| 10 | Apigenin 6,8-C-diglucoside (Vicenin 2) | 4.44 | 1-500 | 0.9998 | 0.477 | 1.444 | 88.84 | 2.37 |
| 11 | Apigenin 6-C-glucoside (Isovitexin) | 5.32 | 1-100 | 0.9998 | 0.577 | 1.748 | 89.80 | 1.79 |
| 12 | Chrysoeriol | 5.42 | 1-100 | 0.9998 | 0.784 | 2.375 | 87.97 | 2.87 |
| 13 | Diosmetin | 7.91 | 1-100 | 0.9998 | 0.579 | 1.753 | 86.90 | 1.83 |
| 14 | Luteolin | 7.06 | 1-100 | 0.9998 | 0.618 | 1.872 | 87.14 | 2.32 |
| 15 | Luteolin 7-O-glucoside (Cynaroside) | 5.42 | 1-100 | 0.9998 | 0.550 | 1.667 | 84.84 | 2.02 |
| 16 | Scutellarein | 6.56 | 1-100 | 0.9998 | 0.709 | 2.149 | 86.96 | 1.47 |
|  | *Flavonols* |  |  |  |  |  |  |  |
| 17 | Isorhamnetin | 7.88 | 1-100 | 0.9999 | 0.388 | 1.176 | 85.13 | 1.95 |
| 18 | Isorhamnetin 3-O-rutinoside (Narcissoside) | 5.85 | 1-1000 | 0.9998 | 0.465 | 1.408 | 86.28 | 2.03 |
| 19 | Kaempferide | 9.28 | 1-100 | 0.9998 | 0.592 | 1.792 | 84.40 | 2.56 |
| 20 | Kaempferol | 7.70 | 1-1000 | 0.9999 | 0.284 | 0.859 | 87.60 | 0.42 |
| 21 | Kaempferol 3-O-glucoside (Astragalin) | 5.90 | 1-200 | 0.9998 | 0.474 | 1.436 | 84.08 | 1.46 |
| 22 | Kaempferol 3-O-rhamnoside (Afzelin) | 6.43 | 1-100 | 0.9998 | 0.542 | 1.641 | 87.33 | 2.00 |
| 23 | Kaempferol 3-O-rutinoside (Nicotiflorin) | 5.74 | 1-1000 | 0.9999 | 0.313 | 0.948 | 85.93 | 2.98 |
| 24 | Kaempferol 3-O-sambubioside | 5.35 | 1-100 | 0.9998 | 0.634 | 1.920 | 84.87 | 2.29 |
| 25 | Quercetin | 6.96 | 1-200 | 0.9998 | 0.500 | 1.515 | 87.36 | 1.44 |
| 26 | Quercetin 3-O-glucoside (Hirsutrin) | 5.46 | 5-1000 | 0.9998 | 0.604 | 1.830 | 89.68 | 1.55 |
| 27 | Quercetin 3-O-malonylglucoside | 5.64 | 10-1000 | 0.9999 | 0.476 | 1.441 | 89.29 | 1.70 |
| 28 | Quercetin 3-O-rhamnoside (Quercitrin) | 5.92 | 1-100 | 0.9998 | 0.673 | 2.039 | 88.04 | 1.92 |
| 29 | Quercetin 3-O-rutinoside (Rutin) | 5.32 | 1-1000 | 0.9999 | 0.379 | 1.148 | 89.16 | 1.94 |
|  | *Flavanones* |  |  |  |  |  |  |  |
| 30 | Eriodictyol | 6.65 | 1-100 | 0.9999 | 0.498 | 1.509 | 87.98 | 2.77 |
| 31 | Naringenin | 7.33 | 1-100 | 0.9997 | 0.792 | 2.400 | 88.88 | 2.70 |
|  | *Flavanols* |  |  |  |  |  |  |  |
| 32 | Catechin | 4.09 | 5-1000 | 0.9999 | 0.327 | 0.990 | 80.74 | 2.65 |
| 33 | Epicatechin | 4.50 | 2-500 | 0.9998 | 0.641 | 1.940 | 81.52 | 2.06 |

Table S4. Metabolite profiles in Asteraceae edible plants from Ulleungdo Island

|  | Compound | Formula | ppm | Calc. MW | *m/z* | RT (min) | Adduct | Major Fragment Ions (*m/z*) |
| --- | --- | --- | --- | --- | --- | --- | --- | --- |
|  | *Hydroxybenzoic acid* |  |  |  |  |  |  |  |
| 1 | 2,5-Di-hydroxybenzoic acid (Gentisic acid) | C_7_ H_6_ O_4_ | −1.08 | 154.0257 | 153.0184 | 8.115 | [M−H]^-1^ | 109.0284(100), 108.0205(71), 153.0183(40), 85.0282(6), 110.0317(6), 123.0073(6), 95.0125(6), 81.0332(4), 154.0217(3), 91.9653(1) |
| 2 | Protocatechuic acid | C_7_ H_6_ O_4_ | −1.13 | 154.0257 | 153.0184 | 5.08 | [M−H]^-1^ | 109.0282(100), 153.0183(16), 110.0316(6), 108.0204(5), 81.0332(1), 154.0217(1), 91.0177(1), 109.1489(0), 65.0382(0), 132.5397(0) |
|  | *Hydroxycinnamic acid* |  |  |  |  |  | [M−H]^-1^ |  |
| 3 | 3*-O-*caffeoylquinic acid (Neochlorogenic acid) | C_16_ H_18_ O_9_ | 0.39 | 354.0952 | 353.0879 | 6.992 | [M−H]^-1^ | 191.0556(100), 135.0441(71), 179.0341(51), 353.0883(23), 85.0281(8), 161.0233(5), 134.0361(4), 93.0333(3), 173.0446(3), 111.0441(3) |
| 4 | 3*-O-*coumaroylquinic acid | C_16_ H_18_ O_8_ | 0.91 | 338.1005 | 337.0932 | 13.823 | [M−H]^-1^ | 191.0556(100), 93.0333(29), 119.0491(13), 163.0391(10), 173.0446(10), 87.0075(8), 337.0941(6), 85.0282(6), 67.0175(6), 111.0439(5) |
| 5 | 3*-O-*feruloylquinic acid | C_17_ H_20_ O_9_ | −0.14 | 368.1107 | 367.1034 | 11.287 | [M−H]^-1^ | 134.0363(100), 193.0501(70), 367.1044(11), 117.0333(9), 149.0598(7), 191.0562(4), 111.0438(3), 155.0338(3), 173.0448(2), 93.0331(2) |
| 6 | 4*-O-*caffeoylquinic acid (Cryptochlorogenic acid) | C_16_ H_18_ O_9_ | 0.29 | 354.0952 | 353.0879 | 11.404 | [M−H]^-1^ | 173.0447(100), 135.0441(96), 179.0342(65), 191.0556(63), 93.0334(32), 353.0885(22), 111.044(10), 85.0282(10), 137.0237(6), 155.0339(6) |
| 7 | 4*-O-*coumaroylquinic acid | C_16_ H_18_ O_8_ | 1.11 | 338.1005 | 337.0933 | 14.037 | [M−H]^-1^ | 173.0447(100), 93.0332(39), 119.049(24), 163.0391(20), 111.044(8), 337.0929(7), 137.0234(7), 67.0175(6), 155.034(5), 71.0125(4) |
| 8 | 5*-O-*caffeoylquinic acid (Chlorogenic acid) | C_16_ H_18_ O_9_ | -0.16 | 354.095 | 353.0878 | 10.664 | [M−H]^-1^ | 191.0556(100), 85.0281(8), 353.0855(5), 220.7875(4), 159.5122(4), 83.3047(3), 228.4181(3), 213.0724(3), 269.3395(3), 50.0775(3) |
| 9 | 5*-O-*coumaroylquinic acid | C_16_ H_18_ O_8_ | 0.84 | 338.1005 | 337.0932 | 16.168 | [M−H]^-1^ | 191.0555(100), 85.0281(7), 93.0331(5), 337.094(5), 119.049(2), 87.0074(2), 173.0447(2), 127.0388(2), 111.0437(1), 163.0391(1) |
| 10 | 5*-O-*feruloylquinic acid | C_17_ H_20_ O_9_ | 0.34 | 368.1109 | 367.1036 | 15.682 | [M−H]^-1^ | 173.0447(100), 93.0333(40), 134.0363(34), 193.0502(15), 367.1041(11), 191.0554(11), 111.0439(9), 67.0176(7), 137.0235(6), 155.0342(6) |
| 11 | 3,4-Di*-O-*caffeoylquinic acid | C_25_ H_24_ O_12_ | 0.52 | 516.127 | 515.1198 | 21.544 | [M−H]^-1^ | 173.0447(100), 179.0341(63), 135.0441(52), 191.0556(46), 515.1194(34), 353.0891(22), 93.0333(16), 111.044(6), 137.0231(6), 155.0338(5) |
| 12 | 3,5-Di*-O-*caffeoylquinic acid | C_25_ H_24_ O_12_ | −0.17 | 516.1267 | 515.1194 | 20.974 | [M−H]^-1^ | 191.0554(100), 179.0343(48), 135.0441(48), 353.0888(20), 515.1167(6), 173.045(6), 161.0236(4), 93.0331(3), 85.0281(3), 407.123(3) |
| 13 | 4,5-Di*-O-*caffeoylquinic acid | C_25_ H_24_ O_12_ | −0.06 | 516.1267 | 515.1195 | 21.69 | [M−H]^-1^ | 173.0448(100), 179.0342(78), 135.0441(56), 191.0559(48), 515.1212(33), 353.0888(23), 93.0332(17), 161.0234(6), 137.0235(6), 111.0439(5) |
| 14 | Chlorogenic acid methyl ester | C_17_ H_20_ O_9_ | 1.33 | 368.1112 | 367.1039 | 17.9 | [M−H]^-1^ | 135.0441(100), 179.034(50), 367.1039(42), 161.0233(29), 133.0283(8), 167.5944(5), 140.7671(5), 148.1272(5), 85.0281(4), 193.8505(4) |
|  | *Flavones* |  |  |  |  |  |  |  |
| 15 | 3,7-Dihydroxy-3',4'-dimethoxyflavone | C_17_ H_14_ O_6_ | 0.33 | 314.0791 | 313.0719 | 24.843 | [M−H]^-1^ | 283.0252(100), 298.0484(70), 313.0719(39), 255.0297(15), 163.0024(9), 227.035(8), 117.0329(7), 104.872(7), 167.5007(5), 235.3978(5) |
| 16 | 3,7-Dihydroxy-3',5'-dimethoxyflavone | C_17_ H_14_ O_6_ | 1.59 | 314.0799 | 313.0726 | 25.88 | [M−H]^-1^ | 283.0261(100), 298.049(68), 313.0733(37), 255.0306(17), 163.0029(11), 164.9809(8), 192.2121(7), 66.8519(7), 294.1541(6), 95.0456(6) |
| 17 | Acacetin | C_16_ H_12_ O_5_ | 1.18 | 284.0688 | 283.0615 | 30.266 | [M−H]^-1^ | 268.0378(100), 283.0616(59), 239.0345(3), 240.0425(3), 151.0018(2), 267.0289(1), 200.0458(1), 212.0476(1), 128.2236(1), 268.7077(1) |
| 18 | Acaciin | C_28_ H_32_ O_14_ | 0.87 | 592.1797 | 591.1724 | 23.911 | [M−H]^-1^ | 283.0616(100), 268.038(81), 87.4412(1), 233.4889(1), 74.317(1), 168.0695(1), 205.3116(1), 93.1856(1), 59.4813(1), 98.2647(1) |
| 19 | Apigenin | C_15_ H_10_ O_5_ | 1.22 | 270.0532 | 269.0459 | 25.906 | [M−H]^-1^ | 269.046(100), 117.0334(26), 151.0028(13), 149.0235(11), 107.0126(6), 225.0555(5), 65.0019(3), 201.0551(3), 227.0347(3), 159.0444(2) |
| 20 | Apigenin 6,8-C-diglucoside (Vicenin 2) | C_27_ H_30_ O_15_ | 1.68 | 594.1595 | 593.1522 | 15.489 | [M−H]^-1^ | 593.1523(100), 353.0673(74), 383.0778(45), 297.0775(42), 473.1097(29), 325.0724(22), 365.0681(13), 323.0559(9), 328.059(8), 295.0611(8) |
| 21 | Apigenin 6-C-arabinoside-8-C-glucoside (Isoschaftoside) | C_26_ H_28_ O_14_ | 2.37 | 564.1492 | 563.142 | 17.019 | [M−H]^-1^ | 563.1351(100), 383.0765(74), 353.0674(63), 297.0769(48), 91.9579(41), 312.264(25), 72.623(23), 278.7149(21), 101.4934(19), 139.9939(19) |
| 22 | Apigenin 6-C-glucoside-8-C-arabinoside (Schaftoside) | C_26_ H_28_ O_14_ | 1.04 | 564.1485 | 563.1412 | 17.697 | [M−H]^-1^ | 563.1423(100), 353.0677(48), 383.0786(31), 297.0771(24), 443.1011(21), 473.1105(12), 325.0719(10), 323.0548(9), 365.0673(8), 328.0583(7) |
| 23 | Apigenin 6-C-rhamnosylglucoside | C_27_ H_30_ O_14_ | 2.25 | 578.1649 | 577.1576 | 19.893 | [M−H]^-1^ | 577.155(100), 353.0659(53), 383.0777(53), 297.0773(35), 91.9653(19), 278.0663(15), 242.9622(14), 278.8848(13), 119.4449(13), 192.2869(13) |
| 24 | Apigenin 7*-O-*glucoside (Apigetrin) | C_21_ H_20_ O_10_ | 0.34 | 432.1058 | 431.0985 | 20.284 | [M−H]^-1^ | 269.0458(100), 431.0986(30), 117.0331(5), 225.0549(5), 183.0446(3), 151.0026(3), 107.0124(3), 149.0234(3), 91.9574(2), 201.0551(2) |
| 25 | Apigenin 7*-O-*rutinoside (Isorhoifolin) | C_27_ H_30_ O_14_ | 0.46 | 578.1638 | 577.1566 | 21.016 | [M−H]^-1^ | 269.0459(100), 577.1577(15), 268.0377(5), 91.9643(2), 117.0332(2), 404.6843(1), 225.0555(1), 234.6838(1), 135.2703(1), 260.8122(1) |
| 26 | Apigenin 8-C-glucoside (Vitexin) | C_21_ H_20_ O_10_ | 2.01 | 432.1065 | 431.0992 | 19.215 | [M−H]^-1^ | 311.057(100), 283.0614(92), 431.0969(49), 341.0645(9), 163.0395(9), 427.5163(8), 429.3889(8), 451.3099(7), 117.033(7), 165.1889(7) |
| 27 | Apigenin-6-C-glucoside (Isovitexin) | C_21_ H_20_ O_10_ | 1.16 | 432.1062 | 431.0989 | 21.449 | [M−H]^-1^ | 268.0383(100), 431.0965(53), 269.0456(16), 78.8475(8), 140.0185(8), 376.2347(8), 143.4813(7), 94.8351(7), 117.99(7), 54.5111(7) |
| 28 | Chrysoeriol | C_16_ H_12_ O_6_ | −0.12 | 300.0634 | 299.0561 | 23.856 | [M−H]^-1^ | 284.0332(100), 299.0564(39), 165.9893(11), 109.9995(8), 150.0246(7), 98.6322(7), 302.0358(6), 86.224(5), 209.2206(5), 101.2781(5) |
| 29 | Chrysoeriol 6-C-glucoside | C_22_ H_22_ O_11_ | 1.06 | 462.1167 | 461.1094 | 21.85 | [M−H]^-1^ | 283.0253(100), 461.1098(71), 297.0407(20), 255.03(15), 284.0332(11), 163.0025(10), 285.0416(8), 298.0482(8), 269.0458(7), 299.0557(7) |
| 30 | Chrysoeriol-5*-O-*glucoside | C_22_ H_22_ O_11_ | 1.93 | 462.1171 | 461.1098 | 20.858 | [M−H]^-1^ | 299.0568(100), 284.0335(65), 461.11(64), 285.0401(10), 394.437(9), 153.7914(9), 98.9845(8), 60.4173(8), 82.6602(8), 62.9215(8) |
| 31 | Chrysoeriol-8-C-glucoside | C_22_ H_22_ O_11_ | 1.4 | 462.1169 | 461.1096 | 20.194 | [M−H]^-1^ | 299.0559(100), 297.041(79), 461.109(72), 298.0477(61), 283.0247(60), 255.0298(57), 284.0321(45), 91.9577(32), 269.0468(24), 117.6063(15) |
| 32 | Cirsimaritin 4′*-O-*glucoside (Cirsimarin) | C_23_ H_24_ O_11_ | 0.66 | 476.1322 | 475.1249 | 24.843 | [M−H]^-1^ | 297.041(100), 313.0724(60), 298.0488(49), 283.0251(42), 269.0457(37), 475.1238(35), 254.0216(15), 254.0585(7), 460.1004(5), 299.0562(4) |
| 33 | Diosmetin | C_16_ H_12_ O_6_ | 1.00 | 300.0637 | 299.0564 | 26.299 | [M−H]^-1^ | 284.0332(100), 299.0568(24), 136.987(22), 65.0019(8), 212.0473(6), 200.0477(4), 283.0256(4), 227.0342(3), 186.0319(3), 228.0428(3) |
| 34 | Diosmetin 7*-O-*neohesperidoside (Neodiosmin) | C_28_ H_32_ O_15_ | 1.26 | 608.1749 | 607.1676 | 22.96 | [M−H]^-1^ | 299.0567(100), 284.0329(76), 607.1699(9), 136.987(6), 343.911(4), 431.6499(3), 68.644(3), 266.2831(3), 60.429(3) |
| 35 | Diosmetin 7*-O-*rutinoside (Diosmin) | C_28_ H_32_ O_15_ | 1.78 | 608.1752 | 607.1679 | 21.443 | [M−H]^-1^ | 284.0329(100), 299.0564(68), 91.9575(15), 607.1657(14), 283.0252(9), 450.5378(7), 277.3177(7), 57.3652(7), 57.8944(6), 86.3993(6) |
| 36 | Hesperetin-5*-O-*glucoside | C_22_ H_24_ O_11_ | 1.41 | 464.1325 | 463.1252 | 21.229 | [M−H]^-1^ | 301.0722(100), 286.0485(69), 165.9897(47), 463.1247(37), 119.049(13), 211.9326(7), 164.9817(7), 180.7492(7), 260.1702(6), 168.1548(5) |
| 37 | Hispidulin | C_16_ H_12_ O_6_ | −1.84 | 300.0622 | 299.055 | 22.96 | [M−H]^-1^ | 284.0332(100), 299.0565(86), 136.9868(27), 283.0244(8), 212.0477(8), 65.0018(8), 256.036(5), 227.0341(5), 200.0472(4), 255.0306(4) |
| 38 | Hispidulin-7*-O-*glucoside | C_22_ H_22_ O_11_ | 2.49 | 462.1174 | 461.1101 | 23.397 | [M−H]^-1^ | 299.0566(100), 284.0331(76), 461.1078(9), 136.9868(7), 461.2427(6), 200.0458(5), 164.5278(5), 92.1493(5), 83.4673(4), 92.4184(4) |
| 39 | Isothymusin | C_17_ H_14_ O_7_ | 2.47 | 330.0748 | 329.0675 | 26.377 | [M−H]^-1^ | 299.0201(100), 314.0438(74), 329.0673(61), 271.026(55), 227.0346(17), 161.0228(11), 180.1172(7), 81.4349(7), 85.6643(6), 184.6722(6) |
| 40 | Jaceosidin-7*-O-*glucoside | C_23_ H_24_ O_12_ | 1.73 | 492.1281 | 491.1208 | 22.233 | [M−H]^-1^ | 313.0367(100), 491.1182(74), 298.0119(44), 90.7469(15), 72.7761(15), 136.229(14), 53.1268(14), 50.475(12), 59.566(12), 53.283(11) |
| 41 | Luteolin | C_15_ H_10_ O_6_ | −1.88 | 286.0472 | 287.0545 | 24.276 | [M−H]^-1^ | 287.055(100), 153.0182(4), 288.0577(3), 121.0284(2), 165.0182(2), 185.0598(1), 111.0075(1), 183.0286(1), 213.0538(1), 137.0228(1) |
| 42 | Pectolinarigenin | C_17_ H_14_ O_6_ | 1.91 | 314.0796 | 313.0724 | 30.85 | [M−H]^-1^ | 283.0252(100), 298.0489(64), 313.072(35), 255.03(17), 163.0033(12), 117.0335(6), 227.0343(5), 183.0444(4), 135.0075(4), 210.1029(2) |
| 43 | Pectolinarigenin-7*-O-*rutinoside (Pectolinarin) | C_29_ H_34_ O_15_ | 0.98 | 622.1904 | 621.1831 | 24.121 | [M−H]^-1^ | 283.0254(100), 313.0725(97), 298.0489(84), 255.03(5), 297.0394(4), 91.9578(2), 68.3798(1), 180.2058(1), 107.3694(1), 354.2042(1) |
| 44 | Scutellarein | C_15_ H_10_ O_6_ | 0.91 | 286.048 | 285.0407 | 26.414 | [M−H]^-1^ | 285.0414(100), 191.3648(7), 266.8192(6), 166.0038(6), 111.3728(6), 90.4157(5), 69.8246(5), 70.0242(5) |
| 45 | Tricin 5*-O-*galactoside | C_23_ H_24_ O_12_ | 1.38 | 492.1275 | 493.1341 | 20.991 | [M+H]^+1^ | 331.081(100), 315.05(21), 270.051(6), 316.0582(5), 332.0842(5), 85.029(3), 287.0546(2), 343.1815(2), 455.4847(2), 403.9063(1) |
| 46 | Tricin 5*-O-*glucoside | C_23_ H_24_ O_12_ | 0.91 | 492.1272 | 493.1345 | 21.971 | [M+H]^+1^ | 331.0811(100), 493.134(27), 315.0495(24), 316.0561(7), 270.0516(4), 91.9598(3), 332.0839(2), 226.9324(2), 392.6537(2), 53.0831(2) |
| 47 | Vitexin 2''*-O-*arabinoside | C_26_ H_28_ O_14_ | 0.68 | 564.1483 | 565.1556 | 18.878 | [M+H]^+1^ | 565.1554(100), 547.1447(39), 379.0821(32), 325.0705(30), 295.0605(22), 271.0592(20), 337.071(20), 349.0693(14), 307.0603(14), 391.0799(13) |
|  | *Flavonols* |  |  |  |  |  |  |  |
| 48 | Datiscetin-3*-O-*rutinoside (Datiscin) | C_27_ H_30_ O_15_ | 0.58 | 594.1588 | 593.1515 | 20.725 | [M−H]^-1^ | 285.041(100), 593.152(86), 255.0297(69), 284.0328(69), 227.0347(16), 229.0502(11), 257.0448(8), 256.0365(6), 211.0401(6), 485.8452(6) |
| 49 | Herbacetin 3,8*-O-*diglucoside (Linocinamarin) | C_27_ H_30_ O_17_ | 2.21 | 626.1497 | 625.1424 | 16.775 | [M−H]^-1^ | 300.0282(100), 301.0352(74), 463.0881(37), 625.141(19), 151.0028(18), 462.5842(11), 95.1882(7), 251.3066(5), 98.9526(5), 59.4216(2) |
| 50 | Isorhamnetin | C_16_ H_12_ O_7_ | 1.01 | 316.0586 | 315.0513 | 26.613 | [M−H]^-1^ | 300.028(100), 315.0516(82), 151.0027(24), 107.0126(16), 271.0266(7), 91.9577(6), 148.0159(5), 164.0105(5), 283.0254(5), 124.0153(4) |
| 51 | Isorhamnetin 3*-O-*acetylgalactoside | C_24_ H_24_ O_13_ | 0.26 | 520.1218 | 519.1146 | 22.297 | [M−H]^-1^ | 299.0196(100), 314.0446(100), 519.117(76), 271.0248(67), 315.0529(58), 243.0293(43), 300.0276(29), 255.0303(24), 262.9658(21), 285.0414(14) |
| 52 | Isorhamnetin 3*-O-*acetylglucoside | C_24_ H_24_ O_13_ | 1.08 | 520.1223 | 519.115 | 22.437 | [M−H]^-1^ | 314.0439(100), 519.1152(95), 243.0299(82), 271.0252(69), 285.0408(38), 299.0201(31), 257.0459(25), 270.0174(16), 315.0523(15), 300.0281(10) |
| 53 | Isorhamnetin 3*-O-*glucuronide | C_22_ H_22_ O_13_ | 1.06 | 494.1066 | 493.0993 | 20.198 | [M−H]^-1^ | 315.0151(100), 330.0391(97), 287.0209(68), 493.1006(64), 331.0461(37), 316.0236(27), 274.2988(12), 98.7226(10), 89.2501(10), 91.9672(9) |
| 54 | Isorhamnetin 3*-O-*neohespeidoside | C_28_ H_32_ O_16_ | 1.37 | 624.1699 | 623.1626 | 20.575 | [M−H]^-1^ | 299.0202(100), 623.1644(76), 271.0262(67), 314.0427(64), 91.9648(24), 272.0326(22), 315.05(20), 300.0275(15), 164.9815(14), 255.0295(13) |
| 55 | Isorhamnetin 3*-O-*rutinoside (Narcissoside) | C_28_ H_32_ O_16_ | 0.60 | 624.1694 | 623.1621 | 21.034 | [M−H]^-1^ | 315.0515(100), 623.1627(69), 299.0201(61), 300.0281(48), 271.0251(44), 314.044(43), 243.0293(29), 255.0298(22), 285.0413(10), 91.9648(5) |
| 56 | Isorhamnetin-3*-O-*arabinoside | C_21_ H_20_ O_11_ | 1.78 | 448.1014 | 447.0941 | 22.069 | [M−H]^-1^ | 314.0443(100), 447.0941(82), 271.0246(60), 243.0301(59), 285.0416(36), 257.044(21), 299.0205(15), 315.0512(10), 242.022(10), 252.2798(7) |
| 57 | Isorhamnetin-3*-O-*malonylglucoside | C_25_ H_24_ O_15_ | 0.70 | 564.1119 | 563.1046 | 22.298 | [M−H]^-1^ | 299.0201(100), 315.0519(79), 314.0437(70), 519.1151(66), 271.0247(50), 300.0284(38), 163.039(24), 255.03(23), 243.03(19), 91.9647(15) |
| 58 | Kaempferide | C_16_ H_12_ O_6_ | 1.50 | 300.0641 | 299.0569 | 26.409 | [M−H]^-1^ | 284.0331(100), 299.056(38), 256.0378(21), 136.9869(7), 65.0019(4), 83.1679(3), 70.157(3), 72.9504(3), 54.7352(3), 74.242(3) |
| 59 | Kaempferol | C_15_ H_10_ O_6_ | 0.56 | 286.0479 | 285.0406 | 26.157 | [M−H]^-1^ | 285.0409(100), 93.0334(3), 185.0603(3), 229.0499(3), 187.0402(2), 151.0026(2), 107.0124(2), 171.0442(2), 239.0343(2), 159.0441(1) |
| 60 | Kaempferol 3,7-Di*-O-*glucoside | C_27_ H_30_ O_16_ | 2.30 | 610.1548 | 609.1475 | 14.883 | [M−H]^-1^ | 283.0251(100), 285.0408(83), 609.1451(35), 255.0297(29), 446.0842(20), 447.0943(15), 596.0694(12), 179.2791(12), 401.9348(11), 134.1534(11) |
| 61 | Kaempferol 3*-O-*arabinoside (Juglalin) | C_20_ H_18_ O_10_ | 1.42 | 418.0906 | 417.0833 | 21.453 | [M−H]^-1^ | 284.0331(100), 255.03(95), 227.0348(68), 417.0833(66), 285.0407(17), 256.0389(8), 229.051(3), 257.0455(3), 417.2112(2), 198.7339(1) |
| 62 | Kaempferol 3*-O-*galactoside (Trifolin) | C_21_ H_20_ O_11_ | 0.75 | 448.1009 | 447.0936 | 20.601 | [M−H]^-1^ | 284.0333(100), 255.0301(96), 227.0346(81), 447.0941(65), 285.0419(27), 277.5603(7), 377.3508(7), 202.1923(6), 225.2508(6), 69.4054(5) |
| 63 | Kaempferol 3*-O-*glucoside (Astragalin) | C_21_ H_20_ O_11_ | 0.51 | 448.1008 | 447.0935 | 21.074 | [M−H]^-1^ | 255.0299(100), 284.033(83), 447.0941(77), 227.0348(74), 285.0411(27), 256.0375(6), 91.966(4), 445.7786(2), 443.458(2), 444.4867(2) |
| 64 | Kaempferol 3*-O-*malonylgalactoside | C_24_ H_22_ O_14_ | 1.10 | 534.1015 | 533.0943 | 22.157 | [M−H]^-1^ | 284.033(100), 255.03(68), 489.1046(40), 285.0407(34), 227.0344(22), 229.0497(5), 257.0457(4), 406.0903(2), 431.6025(2), 459.1559(2) |
| 65 | Kaempferol 3*-O-*malonylglucoside | C_24_ H_22_ O_14_ | 1.8 | 534.1019 | 533.0946 | 22.747 | [M−H]^-1^ | 284.0329(100), 285.0409(93), 255.0298(87), 489.1043(60), 227.0349(20), 229.0499(16), 257.0446(9), 216.753(8), 71.7594(7), 438.9386(7) |
| 66 | Kaempferol 3*-O-*neohesperidoside | C_27_ H_30_ O_15_ | 2.20 | 594.1598 | 593.1525 | 19.683 | [M−H]^-1^ | 284.0331(100), 255.0298(79), 593.1528(39), 227.0342(25), 285.0417(10), 311.7262(8), 191.3082(7), 106.8743(7), 145.0522(7), 117.4173(6) |
| 67 | Kaempferol 3*-O-*rhamnoside (Afzelin) | C_21_ H_20_ O_10_ | 0.62 | 432.1059 | 431.0986 | 22.833 | [M−H]^-1^ | 255.0296(100), 227.0344(73), 431.098(64), 284.0319(56), 98.2446(18), 215.9546(18), 358.0809(17), 135.6495(16), 423.2601(16), 136.126(15) |
| 68 | Kaempferol 3*-O-*rutinoside (Nicotiflorin) | C_27_ H_30_ O_15_ | 1.19 | 594.1592 | 593.1519 | 21.114 | [M−H]^-1^ | 285.0409(100), 284.0331(73), 593.152(54), 255.0299(53), 227.0347(28), 229.0505(17), 257.0454(10), 256.0371(6), 91.9656(6), 151.0026(5) |
| 69 | Kaempferol 3*-O-*sambubioside | C_26_ H_28_ O_15_ | 1.29 | 580.1436 | 579.1363 | 20.161 | [M−H]^-1^ | 284.0329(100), 255.0302(76), 579.138(58), 227.0346(30), 285.0401(22), 458.6273(7), 408.5534(7), 273.994(7), 138.5414(7), 109.9285(6) |
| 70 | Kaempferol-3*-O-*coumarylglucoside (Tiliroside) | C_30_ H_26_ O_13_ | 1.94 | 594.1391 | 593.1318 | 23.929 | [M−H]^-1^ | 285.0404(100), 284.0331(89), 593.1328(81), 255.0291(74), 227.0346(28), 229.0504(22), 91.966(22), 199.6141(12), 163.2998(12), 61.1199(11) |
| 71 | Kaempferol-3*-O-*glucorhamnoside | C_27_ H_30_ O_15_ | 1.32 | 594.1593 | 593.152 | 20.197 | [M−H]^-1^ | 284.0327(100), 255.0300(61), 593.1514(57), 285.0415(38), 227.0337(34), 91.9576(13), 148.9957(8), 103.7216(8), 73.8525(8), 386.5893(8) |
| 72 | Mearnsetin 3*-O-*rhamnoside (Mearnsitrin) | C_22_ H_22_ O_12_ | 0.43 | 478.1113 | 477.1041 | 21.405 | [M−H]^-1^ | 271.0249(100), 299.02(90), 477.1054(83), 314.0438(74), 315.0517(34), 300.0284(29), 243.0288(23), 272.0321(11), 255.0305(9), 165.9898(7) |
| 73 | Myricetin 3*-O-*rhamnoside (Myricitrin) | C_21_ H_20_ O_12_ | 1.7 | 464.0963 | 463.089 | 19.492 | [M−H]^-1^ | 300.0277(100), 271.0243(67), 463.0919(43), 255.03(27), 301.0349(25), 243.0288(17), 151.0025(11), 424.6345(11), 188.9272(10), 382.2798(10) |
| 74 | Myricetin 3*-O-*rutinoside | C_27_ H_30_ O_17_ | 1.64 | 626.1493 | 625.1421 | 17.199 | [M−H]^-1^ | 300.0281(100), 301.0355(92), 463.091(30), 151.0029(29), 271.0243(20), 255.0314(16), 252.8096(11), 224.0556(10), 317.3657(10), 127.9323(8) |
| 75 | Patuletin-3*-O-*rutinoside | C_28_ H_32_ O_17_ | 1.57 | 640.165 | 639.1577 | 19.829 | [M−H]^-1^ | 330.0387(100), 315.0162(80), 639.1575(75), 287.0199(53), 331.0459(39), 566.349(17), 307.0896(15), 149.0246(14), 52.6427(14), 174.9998(13) |
| 76 | Quercetin | C_15_ H_10_ O_7_ | 1.15 | 302.043 | 301.0357 | 24.087 | [M−H]^-1^ | 151.0027(100), 301.0357(97), 107.0126(39), 178.9979(37), 121.0283(35), 65.0018(14), 83.0125(11), 63.0228(8), 93.0332(6), 68.9135(5) |
| 77 | Quercetin 3,7*-O-*diglucoside | C_27_ H_30_ O_17_ | 1.7 | 626.1494 | 625.1421 | 13.062 | [M−H]^-1^ | 299.0204(100), 301.0356(45), 625.1436(44), 271.0244(34), 462.0832(30), 77.6678(13), 107.1212(13), 119.4979(13), 402.0271(13), 177.2506(12) |
| 78 | Quercetin 3*-O-*Coumaroylgalactoside | C_30_ H_26_ O_14_ | 1.51 | 610.1338 | 609.1265 | 23.295 | [M−H]^-1^ | 300.0275(100), 301.0353(55), 609.1296(49), 271.0242(40), 463.089(36), 91.9579(26), 255.0304(21), 151.0031(20), 438.9136(13), 243.0618(12) |
| 79 | Quercetin 3*-O-*glucoside (Hirsutrin) | C_21_ H_20_ O_12_ | 1.09 | 464.096 | 463.0887 | 19.783 | [M−H]^-1^ | 300.0277(100), 271.025(74), 463.0887(58), 301.0356(35), 255.0297(28), 151.0027(15), 243.0293(9), 178.9979(5), 163.0032(4), 107.0125(3) |
| 80 | Quercetin 3*-O-*malonylgalatoside | C_23_ H_22_ O_13_ | 0.95 | 506.1065 | 505.0992 | 20.58 | [M−H]^-1^ | 300.0282(100), 271.0255(66), 255.0304(34), 505.0999(33), 301.035(18), 243.029(11), 151.0025(9), 91.9588(7), 166.9164(6), 472.1957(6) |
| 81 | Quercetin 3*-O-*malonylglucoside | C_23_ H_22_ O_13_ | 1.46 | 506.1068 | 505.0995 | 21.358 | [M−H]^-1^ | 300.0275(100), 271.0251(67), 505.0998(41), 255.0297(30), 301.0351(18), 151.0028(13), 91.965(12), 238.4418(6), 98.7616(5), 61.0849(5) |
| 82 | Quercetin 3*-O-*rhamnoside (Quercitrin) | C_21_ H_20_ O_11_ | 1.44 | 448.1012 | 447.0939 | 21.458 | [M−H]^-1^ | 300.0287(100), 447.0925(60), 271.0246(57), 255.0291(36), 301.0346(17), 285.0406(12), 345.3761(12), 238.8118(11), 332.8159(11), 69.0594(11) |
| 83 | Quercetin 3*-O-*rutinoside (Rutin) | C_27_ H_30_ O_16_ | 0.66 | 610.1538 | 609.1465 | 19.461 | [M−H]^-1^ | 300.0279(100), 609.1466(60), 271.0252(50), 301.0356(30), 255.0297(24), 151.0027(10), 178.9975(7), 243.03(6), 254.0208(4), 163.0035(3) |
|  | *Flavanones* |  |  |  |  |  | [M−H]^-1^ |  |
| 84 | Eriodictyol-3'*-O-*glucoside | C_21_ H_22_ O_11_ | 1.68 | 450.117 | 449.1097 | 16.092 | [M−H]^-1^ | 151.0027(100), 135.044(77), 287.0568(29), 449.1056(26), 107.0125(16), 205.7481(9), 354.6559(9), 87.3137(8), 189.262(8), 125.0238(8) |
| 85 | Eriodictyol-7*-O-*malonylglucoside | C_24_ H_24_ O_14_ | 1.58 | 536.1175 | 535.1102 | 15.543 | [M−H]^-1^ | 269.0459(100), 225.0555(23), 197.0603(22), 493.1354(18), 350.0928(18), 547.508(17), 76.1872(15), 98.5952(15), 73.8299(15), 91.9689(15) |
| 86 | Naringenin | C_15_ H_12_ O_5_ | 1.87 | 272.069 | 271.0617 | 25.536 | [M−H]^-1^ | 119.049(100), 271.0616(86), 151.0029(85), 107.0127(43), 65.0018(20), 93.0335(11), 177.019(10), 63.0228(10), 173.5674(8), 59.3038(8) |
| 87 | Naringenin-4'*-O-*galactoside | C_21_ H_22_ O_10_ | 1.44 | 434.1219 | 433.1147 | 22.543 | [M−H]^-1^ | 151.0027(100), 271.0615(82), 119.0492(61), 107.0125(27), 433.115(21), 351.4494(10), 165.0183(10), 360.5417(9), 276.6306(8), 205.72(8) |
| 88 | Naringenin-4'*-O-*glucoside | C_21_ H_22_ O_10_ | 2.06 | 434.1222 | 433.1149 | 22.723 | [M−H]^-1^ | 271.062(100), 151.0026(90), 119.0492(62), 107.0125(45), 433.1131(34), 65.0019(19), 222.2145(12), 165.0176(12), 247.2683(11), 83.0123(10) |
| 89 | Naringenin-5*-O-*galactoside | C_21_ H_22_ O_10_ | 1.43 | 434.1219 | 433.1146 | 18.146 | [M−H]^-1^ | 271.0614(100), 151.0027(88), 119.0492(32), 433.1169(28), 107.0125(17), 83.0124(10), 177.0188(8), 398.1172(7), 424.0983(6), 310.0174(6) |
| 90 | Naringenin-5*-O-*glucoside | C_21_ H_22_ O_10_ | 0.38 | 434.1215 | 433.1142 | 18.986 | [M−H]^-1^ | 271.0614(100), 151.0027(88), 119.049(39), 433.1135(37), 107.0127(20), 83.0125(7), 145.0282(7), 93.0331(7), 165.0181(6), 227.0701(6) |
| 91 | Naringenin-7*-O-*galactoside | C_21_ H_22_ O_10_ | 1.79 | 434.1221 | 433.1148 | 21.145 | [M−H]^-1^ | 271.0617(100), 151.0028(60), 433.1098(25), 119.0492(24), 107.0126(13), 447.8915(12), 254.5595(12), 240.7358(11), 154.8094(11), 54.9153(11) |
|  | *Flavanols* |  |  |  |  |  | [M−H]^-1^ |  |
| 92 | Epicatechin | C_15_ H_14_ O_6_ | 0.75 | 290.0793 | 289.072 | 13.692 | [M−H]^-1^ | 289.0723(100), 109.0282(75), 123.0439(56), 245.0817(48), 125.0233(46), 203.0711(39), 151.0391(26), 205.0506(24), 137.0234(24), 97.0281(22) |
|  | *Anthocyanins* |  |  |  |  |  |  |  |
| 93 | Cyanidin-3*-O-*glucoside | C_21_ H_20_ O_11_ | −0.11 | 448.1005 | 449.1078 | 12.346 | [M+H]^+1^ | 287.0549(100), 449.1074(23), 449.0477(2), 449.1684(2), 357.1077(1), 147.8942(1), 148.5419(1), 116.8668(1), 98.5976(1), 74.9836(1) |
|  | *Isoflavones* |  |  |  |  |  |  |  |
| 94 | Glycitein | C_16_ H_12_ O_5_ | −1.52 | 284.068 | 283.0608 | 23.921 | [M-H]^-1^ | 268.0381(100), 283.0618(51), 240.0437(4), 239.0342(3), 151.0023(2), 91.9655(2), 267.0301(2), 211.0393(1), 107.012(1), 212.0465(1) |
| 95 | Tectorigenin 7*-O-*glucoside (Tectoridin) | C_22_ H_22_ O_11_ | 1.45 | 462.1169 | 461.1096 | 20.574 | [M-H]^-1^ | 283.0253(100), 461.1093(45), 284.0332(39), 255.0303(32), 299.0566(18), 251.9196(5), 122.2418(4), 296.7882(4), 312.957(4), 109.2602(4) |
|  | *Chalcones* |  |  |  |  |  |  |  |
| 96 | 2',4-Dihydroxy-3-methoxychalcone | C_16_ H_14_ O_4_ | 1.19 | 270.0895 | 269.0823 | 29.96 | [M−H]^-1^ | 269.0821(100), 134.0362(79), 137.0233(49), 178.0263(27), 161.0234(24), 133.0282(23), 139.0387(14), 106.0409(8), 62.9626(8), 168.8352(8) |
| 97 | Okanin-4′*-O-*glucoside (Marein) | C_21_ H_22_ O_11_ | 2.01 | 450.1171 | 449.1098 | 21.455 | [M−H]^-1^ | 151.0026(100), 135.0441(71), 287.0569(33), 449.1098(23), 107.0128(12), 239.6509(4), 118.2604(4), 124.2666(4), 249.9804(4), 216.2553(4) |
| 98 | Phloretin 2′*-O-*acetylglucoside | C_23_ H_26_ O_11_ | 0.38 | 478.1477 | 477.1404 | 21.7 | [M−H]^-1^ | 123.044(100), 167.0341(62), 108.0205(46), 477.1385(11), 329.5641(3), 83.3744(3), 115.4034(3), 114.7576(3), 234.35(3), 60.9989(3) |
| 99 | Phloretin 4*-O-*glucoside (Trilobatin) | C_21_ H_24_ O_10_ | 0.16 | 436.137 | 435.1297 | 20.302 | [M−H]^-1^ | 123.044(100), 167.0341(63), 108.0205(44), 435.1299(8), 81.0711(1), 108.013(1), 318.5755(1), 102.5112(1), 59.0124(1), 68.8021(1) |
| 100 | Phloretin-2′*-O-*glucoside (Phloridzin) | C_21_ H_24_ O_10_ | 0.52 | 436.1372 | 435.1299 | 24.435 | [M−H]^-1^ | 123.0441(100), 167.0343(68), 108.0205(46), 435.1313(10), 91.9645(2), 297.5353(1), 311.5788(1), 138.8729(1), 199.0262(1), 163.209(1) |
|  | *Coumarin* |  |  |  |  |  |  |  |
| 101 | Esculin | C_15_ H_16_ O_9_ | 1.04 | 340.0798 | 339.0725 | 8.399 | [M−H]^-1^ | 177.0185(100), 339.0711(13), 133.0286(7), 289.2202(5), 150.4873(4), 246.3972(4), 156.3658(4), 71.2533(4), 117.8879(3), 76.5366(3) |
|  | *Aurone* |  |  |  |  |  |  |  |
| 102 | Maesopsin | C_15_ H_12_ O_6_ | 2.37 | 288.0641 | 287.0568 | 23.493 | [M−H]^-1^ | 135.0442(100), 151.0029(54), 107.0128(22), 65.0019(10), 287.0555(9), 83.0128(7), 150.9917(4), 161.1514(3), 198.314(3), 181.0004(3) |
|  | *Phenylpropanoid* |  |  |  |  |  |  |  |
| 103 | Syringin | C_17_ H_24_ O_9_ | −0.95 | 372.1417 | 373.1493 | 11.392 | [M+H]^+1^ | 395.1313(100), 232.0708(10), 233.0785(6), 185.0427(3), 364.1161(2), 396.1318(2), 148.9835(1), 229.2643(1), 218.1463(1), 105.5037(1) |
|  | *Phenylethanoid* |  |  |  |  |  |  |  |
| 104 | Hydroxytyrosol 4*-O-*glucoside | C_14_ H_20_ O_8_ | 0.75 | 316.1161 | 315.1088 | 5.712 | [M−H]^-1^ | 315.1091(100), 135.0441(97), 71.0124(67), 89.0232(58), 153.0548(47), 123.044(41), 101.023(39), 119.0336(21), 113.0231(18), 92.4184(2) |
|  | *Glucosinolates* |  |  |  |  |  |  |  |
| 105 | 10-Methylsulphinyl-decanenitrile | C_11_ H_21_ N O S | 0.96 | 215.1346 | 216.1419 | 24.553 | [M+H]^+1^ | 216.1419(100), 152.1435(30), 93.0705(10), 96.0813(9), 107.086(9), 65.0063(8), 110.0969(7), 69.0706(6), 82.0657(5), 79.0549(5) |
| 106 | 11-Methylsulphinyl-undecanenitrile | C_12_ H_23_ N O S | 0.33 | 229.1501 | 230.1574 | 26.912 | [M+H]^+1^ | 230.1575(100), 166.1595(23), 110.0967(10), 93.0702(9), 96.0812(9), 124.112(6), 81.0706(5), 79.0549(5), 107.0861(5), 67.0551(4) |
| 107 | 7-Methylsulfinylheptyl isothiocyanate | C_9_ H_17_ N O S_2_ | 0.46 | 219.0753 | 220.0825 | 26.749 | [M+H]^+1^ | 156.0843(100), 220.0826(95), 55.055(42), 123.1045(36), 97.1017(32), 69.0706(12), 147.0838(10), 65.0063(9), 114.0377(8), 95.0862(8) |
| 108 | 8-Methylsulfinyloctyl isothiocyanate | C_10_ H_19_ N O S_2_ | -0.42 | 233.0907 | 234.098 | 28.987 | [M+H]^+1^ | 234.0982(100), 170.0999(79), 69.0706(71), 137.1201(14), 114.0376(14), 161.0996(10), 55.055(8), 65.0064(7), 108.0811(7), 128.0531(6) |
| 109 | 9-Methylsulfinyl-nonanenitrile | C_10_ H_19_ N O S | 1.12 | 201.119 | 202.1262 | 21.744 | [M+H]^+1^ | 202.1262(100), 138.1279(37), 93.0704(21), 65.0063(9), 82.0657(9), 96.0813(9), 121.1014(8), 79.0549(7), 95.086(6), 81.0705(5) |
| 110 | 9-Methylsulfinylnonyl isothiocyanate | C_11_ H_21_ N O S_2_ | 0.45 | 247.1066 | 248.1138 | 31.032 | [M+H]^+1^ | 248.114(100), 184.1158(65), 69.0706(47), 83.0862(23), 114.0377(15), 55.0551(13), 65.0062(9), 128.0532(9), 151.1353(9), 175.1153(7) |

Figure S1. Calibration curves of phenolic compounds used for targeted quantification.

(A)

(B)

(C)

(D)

(E)

(F)

Figure S2. Negative mode of total ion chromatogram (A: freeze-dried *Aster pseudoglehnii* (FAP), B: freeze-dried *Cirsium nipponicum* (FCN), C: freeze-dried *Solidago virgaurea* (FSV), D: sun-dried *Aster pseudoglehnii* (SAP), E: sun-dried *Cirsium nipponicum* (SCN), F: sun-dried *Solidago virgaurea* (SSV))

(A)

(B)

(C)

(D)

(E)

(F)

Figure S3. Positive mode of total ion chromatogram (A: freeze-dried *Aster pseudoglehnii* (FAP), B: freeze-dried *Cirsium nipponicum* (FCN), C: freeze-dried *Solidago virgaurea* (FSV), D: sun-dried *Aster pseudoglehnii* (SAP), E: sun-dried *Cirsium nipponicum* (SCN), F: sun-dried *Solidago virgaurea* (SSV))

(A)

(B)

Figure S4. Internal validation of the Random Forest classification model. (A) Confusion matrix obtained using leave-one-out cross-validation (LOOCV). (B) Distribution of classification accuracy from 1,000 label permutations.
